# Children in non-clinical healthcare environment: insights from a multi-method qualitative approach

**DOI:** 10.1080/17482631.2026.2721235

**Published:** 2026-08-27

**Authors:** Charline Stella Samuel, Nandineni Rama Devi

**Affiliations:** a Manipal School of Architecture and Planning, Manipal Academy of Higher Education, Manipal, India

**Keywords:** Built environment, non-clinical environment, child-centered, multi-sensory, multi-method qualitative approach, lived experience

## Abstract

**Purpose:**

Healthcare visits for children are often stressful. They , more than adults, rely on environmental cues to guide their behaviours in common areas. Despite this, healthcare research has largely focused on patient rooms and clinical spaces, with limited attention to non-clinical spaces. This highlights the need for research that explores children's experiences within non-clinical settings. .The study aims to explore children’s behaviours and experiences within non-clinical healthcare settings, using a multi-method qualitative approach informed by Supportive Design Theory.

**Methods:**

Study was carried out in the pediatric department of a general hospital in South India from July to December 2023. Convenience sampling was used for selecting 25 children aged 5–18 years across four common spaces: play area, waiting area, corridor, and lobby. Three qualitative methods – observation, drawing, and verbal elicitation were used. Observational data descriptively analyzed behavioural patterns, while drawings and verbal responses were thematically analyzed using ATLAS.ti. Three themes emerged: (1) *Familiar environmental engagement*, interaction with recognizable features; (2) *Curiosity-driven environmental engagement*, responses to sensory stimuli elements; and (3) *Inspirational environmental engagement*, imaginative and learning-oriented engagements . Triangulating three datasets deepened the qualitative interpretation.

**Results:**

Findings showed children's behavioural variations across healthcare settings. Sensory-rich and visually engaging environments, particularly the play area and waiting area, supported active behaviours, whereas settings with fewer environmental features predominantly passive engagements. Results suggest that visually familiar, , interactive, and curiosity-supporting elements appeared to support children's engagement during hospital visits.

**Conclusion:**

Study highlights the importance of multi-sensory features within non-clinical settings. Consistent with supportive design theory, findings suggest familiar, sensory-rich, and engaging environmental features may support children's experiences during hospital visits. Study contributes to advancing environment-behaviour research in healthcare contexts.

## 1. Introduction

“*The environments of children are not always environments for children: in many cases, the places where children grow up, play, and learn are, at best, designed for them by adults; at worst they are the spaces left over from the ‘adult world’.”* (Spencer & Blades, 2006)

This observation captures a long-standing discourse on how spaces for children were conceived and designed by adults. Although children inhabit environments such as schools, neighbourhoods and hospitals alongside adults, these settings were often structured around adult-centered perspectives. This is particularly evident in healthcare settings, where spatial decisions are primarily guided by clinical demands, technological advances and safety regulations. As a result, children navigate through spaces that feel unfamiliar, overwhelming and at times intimidating, potentially intensifying anxiety and stress during the hospital visits.

Supportive design theory provides an interpretive lens for understanding how environmental features may support children's engagement by reducing stress and support coping through sensory interaction with meaningful environmental stimuli (Ulrich, 1991, 2001; Ulrich et al., 2008). The physical environment plays a significant role in shaping children's emotional responses and behaviours, especially in unfamiliar and stressful contexts (Evans, 2021; Melles et al., 2020; Watts & Wilson, 2009). Environmental qualities such as colour, texture and sound can either heighten stress or support comfort and coping (Hunleth et al., 2022; Melles et al., 2020). While healthcare research has examined stress reduction in such spaces, much of this evidence is drawn from adult populations (Ulrich et al., 2008; Watts & Wilson, 2009). Children, however, perceive and experience the physical environment differently from adults (Ferguson et al., 2013; Piaget, 1950). Their responses to healthcare physical environments (hereafter referred to as HPE) may also be influenced by previous experiences, health-related concerns, and individual developmental characteristics, which can shape how they engage with environmental features (Ulrich et al., 2008; Watts & Wilson, 2009). Their engagement is often closely linked to sensory cues, opportunities for movement, exploration and play (Evans, 2021; Lambert et al., 2014).

Hospitals, however, are frequently perceived by children as sterile, noisy and restrictive environments that may amplify feelings of fear and discomfort in them (Aldridge and Wood, 1997; Watts & Wilson, 2009). Designing healthcare spaces, therefore, involves more than accommodating medical functions; it also requires attention to children's emotional needs and the need for meaningful engagement (Kaelin & Okland, 2018). Recent studies in child-centered healthcare design highlighted the importance of play opportunities, sensory engagement, nature-based features, and supportive environmental attributes in improving children's healthcare experiences and well-being (Babbu & Haque, 2023; Bahrami et al., 2025; Mohd Zairul and Noor Hafizah, 2025; Yu et al., 2024). Evidence-based reviews have further indicated associations between healthcare environment, patient satisfaction, stress reduction, emotional comfort, and pediatric health outcomes (Bock et al., 2021). Similarly, recent expert perspectives have highlighted emerging priorities in pediatric healthcare design, including adaptability, family-centered care, neurodiversity-sensitive environments, and supportive healthcare experiences (Sathyanarayanan & Caldas, 2025). Despite these advances, a notable gap remains in the empirical research that centres children's voices and perceptions in understanding how they engage with non-clinical healthcare settings (Einarsdóttir, 2007; Melles et al., 2020; Sevón et al., 2023; Williams et al., 2022). Recent methodological discussions have increasingly argued for recognising children as capable social actors and involving them more actively in research about their environments (Bock et al., 2021; Clark, 2008). Qualitative child-centered approaches are particularly well suited to this aim, as they are sensitive to developmental differences and allow children to express their feelings in ways that extend beyond verbal language (Einarsdóttir, 2007; Sevón et al., 2023; Williams et al., 2022).

Addressing these gaps, the present study aims to understand how children behaviourally and experientially engage in non-clinical healthcare environments through a multi-method qualitative approach integrating observations, drawings, and verbal elicitations. Drawing on perspectives from environmental psychology, particularly guided by supportive design theory as an interpretive framework, the study investigates how environmental features support different forms of children's engagement within routine healthcare settings (Bock et al., 2021; Ulrich et al., 2008). By foregrounding children’s lived experiences, the study contributes to environment-behaviour research and advances deeper understanding of how everyday healthcare environments shape children’s interaction with space.

## 2. Literature review

### 2.1. Children and their physical environment

Studies highlight the role of physical environment in shaping children's cognitive, emotional, social, and behavioural responses across diverse environments, including homes, schools, playgrounds, and public spaces (Evans, 2021; Ferguson et al., 2013; Spencer & Blades, 2006). Children rely more on environmental cues to guide their actions and interactions within physical spaces than adults (Piaget, 1950). Research in child-environment studies indicates that environmental attributes including scale, colour, layout and opportunities for movement provide children with the affordances for exploration, learning, independence, and sensory engagement (Coyne, 2006; Einarsdóttir, 2007; Spencer & Blades, 2006).

In educational and recreational settings, spatial characteristics such as natural light, greenery, flexible layouts, visual transparency, and access to outdoor areas have been associated with children's learning, creativity, and social interactions (Evans, 2021; Ferguson et al., 2013; Spencer & Blades, 2006). These suggest that physical environments play an important role in shaping how children experience, interpret, and engage with their surroundings.

Children's interaction with their immediate environment varies across developmental age groups and has considerable impact on how they perceive and respond in the environment (Watts & Wilson, 2009). Younger children tend to engage with their surroundings through sensory inputs such as colour, texture, sound, and scale, while older children interpret through cognitive, social and emotional considerations (Einarsdóttir, 2007; Evans, 2021). For instance, younger children are often drawn to vivid visual stimuli and tactile elements, whereas teenagers tend to prefer spaces that offer freedom, support self-expression, and foster peer interaction (Evans, 2021; Ferguson et al., 2013; Lambert et al., 2014; Spencer & Blades, 2006). These age-related developmental variations highlight the importance of considering environmental engagement in light of children's evolving abilities, experiences, and spatial understanding (Spencer & Blades, 2006).

The relationship between children and their physical environment becomes particularly important in healthcare contexts, which are often perceived as intimidating, stressful, and highly controlled (Aldridge and Wood, 1997; Watts & Wilson, 2009). According to Evans (2021), clinical environments characterized by sterility and noise may heighten stress and overwhelm the senses, while familiar objects, soothing colour palettes and sensory-enriching features may enhance comfort and coping (Evans, 2021). Similarly, Bock et al. (2021) found that thoughtfully designed pediatric healthcare spaces are associated with positive patient experiences, lower stress levels, greater emotional comfort, and enhanced health outcomes. Together, these insights suggest that environmental characteristics may shape how children perceive and respond to healthcare settings. The physical setting thus acts as a key mediator of children's emotional and behavioural responses, shaping how they think, behave and relate to space. In general, research shows that well-planned physical environments can support children's development while easing stress and discomfort (Evans, 2021; Ferguson et al., 2013).

Further, more recent work in healthcare design has additionally drawn attention to the need for an environment that balances stimulating, child-appropriate elements and considerations such as adaptability, infection prevention, accessibility, and family-centered approaches to care (Nuvolari-Duodo et al., 2024; Sathyanarayanan & Caldas, 2025). Additional studies have also stressed the importance of inclusive healthcare spaces that accommodate the needs of diverse pediatric populations, including children with disabilities and varying sensory requirements (Goel et al., 2024; Yu et al., 2024).

Taken together, these studies suggest that environmental characteristics may shape how children experience and respond to healthcare settings. Understanding these relationships calls for a theoretical lens capable of explaining how environmental features support engagement, coping, and stress reduction in healthcare contexts.

### 2.2. Supportive design theory as an interpretive lens

Supportive design theory, originally introduced by Ulrich (1991) and further developed through subsequent healthcare design research that offers a framework for understanding how environmental features may shape children's experiences in healthcare settings (Ulrich et al., 2008; Ulrich, 2001). The theory has informed evidence-based healthcare design by highlighting the role of positive distractions, sense of control, and social support in reducing stress and improving healthcare experiences (Evans, 2021; Ferguson et al., 2013). Within this framework, the link between environmental characteristics and children's experiences can be more clearly understood. The theory suggests that environmental features can help reduce stress through positive distraction, sense of control, and opportunities for social support. Positive distractions refer to environmental elements that attract attention away from stress-inducing situations and encourage engagement with more pleasant or meaningful experiences (Ulrich et al., 2008).

The present study is situated within the broader tradition of environment–behaviour research and environmental psychology, both of which examine the reciprocal relationship between individuals and their physical surroundings (Coyne, 2006; Evans, 2021; Ferguson et al., 2013). Within this broader perspective, supportive design theory serves as the primary interpretive framework guiding the understanding of how environmental features may influence children's experiences within healthcare settings (Ulrich, 1991, 2001; Ulrich et al., 2008). While environment–behaviour research and environmental psychology provide the conceptual foundation for examining children's interactions with healthcare environments, SDT offers a more specific lens through which patterns of engagement, positive distraction, and coping may be interpreted (Bock et al., 2021; Ulrich et al., 2008; Ulrich, 2001).

Within pediatric healthcare environments, SDT provides a useful interpretive lens for understanding how children respond to environmental features beyond their functional purpose. Elements such as play opportunities, visual stimuli, sensory features, familiar objects, and interactive installations may serve as positive distractions that encourage exploration, engagement, and coping within otherwise unfamiliar healthcare settings (Bock et al., 2021; Ulrich et al., 2008; Ulrich, 2001). Recent pediatric healthcare research similarly highlights the importance of play, sensory engagement, child-friendly design, and supportive environments in shaping children's healthcare experiences (Bahrami et al., 2025; Sathyanarayanan & Caldas, 2025; Yu et al., 2024). Through this lens, children's behavioural and experiential engagement may be understood as responses to environmental attributes that support comfort, curiosity, and interaction within healthcare environments. While supportive design theory provides a framework for understanding these relationships, investigating how children experience and engage with healthcare environments requires methodological approaches that can capture children's perspectives directly and in developmentally appropriate ways.

### 2.3. Research methods with children

Children communicate and interpret their experiences differently from adults; researchers increasingly advocate for child-centered methods that are sensitive to children's developmental characteristics and preferred modes of expression (Agarwal et al., 2021; Zhang & Wyness, 2024). Literature shows that children's experiences are significantly influenced by their physical environments (Barrett & Twycross, 2018; Bock et al., 2021; Greig et al., 2013). However, it is also important to examine how various studies have documented these experiences. Studies suggest that data collection with children should be tailored to their developmental and cognitive needs (Agarwal et al., 2021; Zhang & Wyness, 2024). Research involving children increasingly encourages observation and art-based methods. Observations allow researchers to understand children's non-verbal behaviours and activities in natural settings (Greig et al., 2013; Rapoport, 1990). Participatory approaches such as drawings, art-based activities, visual prompts or elicitations provide additional opportunities for children to express their thoughts and experiences through creative mediums (Clark, 2008; Zhang & Wyness, 2024). Such approaches are particularly valuable when exploring environmental experiences that may be difficult for children to express verbally. Further, verbal elicitations rely on children's verbal expressions and interpretations using flexible and child-sensitive prompts (Zhang & Wyness, 2024). Young children often respond more naturally to drawing-based or visual approaches, whereas older children may be more comfortable expressing experiences verbally, although there may be some hesitations depending on context and social comfort (Aldridge and Wood, 1997; Clark, 2008; Zhang & Wyness, 2024). This highlights the importance of flexible and developmentally appropriate methodologies for conducting research with children.

While existing research has highlighted the importance of physical environments in shaping children's experiences, much of the existing evidence has focused on specialized pediatric wards, inpatient settings, specific design interventions, or caregiver perspectives (Bock et al., 2021; Watts & Wilson, 2009). Children commonly spend considerable time within environments such as waiting areas, corridors, lobbies, and play areas during routine healthcare visits. These settings form essential spaces for the patient journey, accommodating waiting, movement, and transitions between different stages of care (Goel et al., 2024; Lambert et al., 2014; Ulrich et al., 2008). Nevertheless, despite their relevance, these spaces have received comparatively under-explored within environment–behaviour research, particularly when viewed through the lens of children's own perspectives.

### 2.4. Rationale for the present study

Despite the growing importance of child-friendly healthcare environments, comparatively limited research has explored how children behaviourally and experientially engage with everyday healthcare spaces using child-appropriate approaches. Although studies using one method have offered valuable insights, fewer have combined multiple child-centered approaches to examine their engagement in healthcare environments (Barrett & Twycross, 2018; Hunleth et al., 2022).

To bridge this gap, the present study adopts a multi-method qualitative approach combining behavioural observations, drawings and verbal elicitations. Grounded in supportive design theory, the study examines how children behaviourally and experientially engage in everyday healthcare environments. Integrating these child-centered methods enables a richer understanding of children's experiences within the HPE and enables deeper interpretation through triangulation across complementary data sources (Agarwal et al., 2021; O'Cathain et al., 2010).

## 3. Site context

This study was conducted within a pediatric department of a general hospital in South India from July to December 2023. The research focused on four hospital settings frequently visited by children: the play area, waiting area, corridor, and lobby. The observations were guided by a set framework adapted from Rapoport's conceptualization of the built environment (1990) (Rapoport, 1990), which classifies the physical environment into observable architectural attributes. This framework groups environmental features into three major components: enclosures, fenestrations, and accessories. Enclosures comprise walls, floors, and ceilings; fenestrations include windows, doors, and other openings, while accessories encompass aesthetic elements, furnishings, and fixtures. Each category was further elaborated through observable characteristics such as height, finish, material, colour, geometry, pattern, texture, size, and location. This classification offered a comprehensive structure for documenting the architectural features of the healthcare physical environment (HPE) and examining children's behavioural responses within these settings. Table I summarizes the categorization of the physical environment into observable architectural attributes, while Supplementary Information 1 (SI-1) provides a detailed description of each category and its associated characteristics.

**Table I.** Categorization of physical environment into observable architectural attributes (Rapoport, 1990).

Major category	Enclosures (E)			Fenestrations (F)			Accessories (A)		
Minor category	Wall	Floor	Ceiling	Windows	Doors	Openings	Aesthetics	Furnishing	Fixtures
Characteristics/attributes	Height, finish, colour, geometry, levels, pattern			Type, height, width, material, location			Artwork, installations, materials, texture, colour, size, shape, pattern, location		

Using the classification presented in Table I, the physical attributes of each study setting were systematically documented through direct observations as presented in Table II below.

**Table II.** Physical attributes documented at the four study settings.

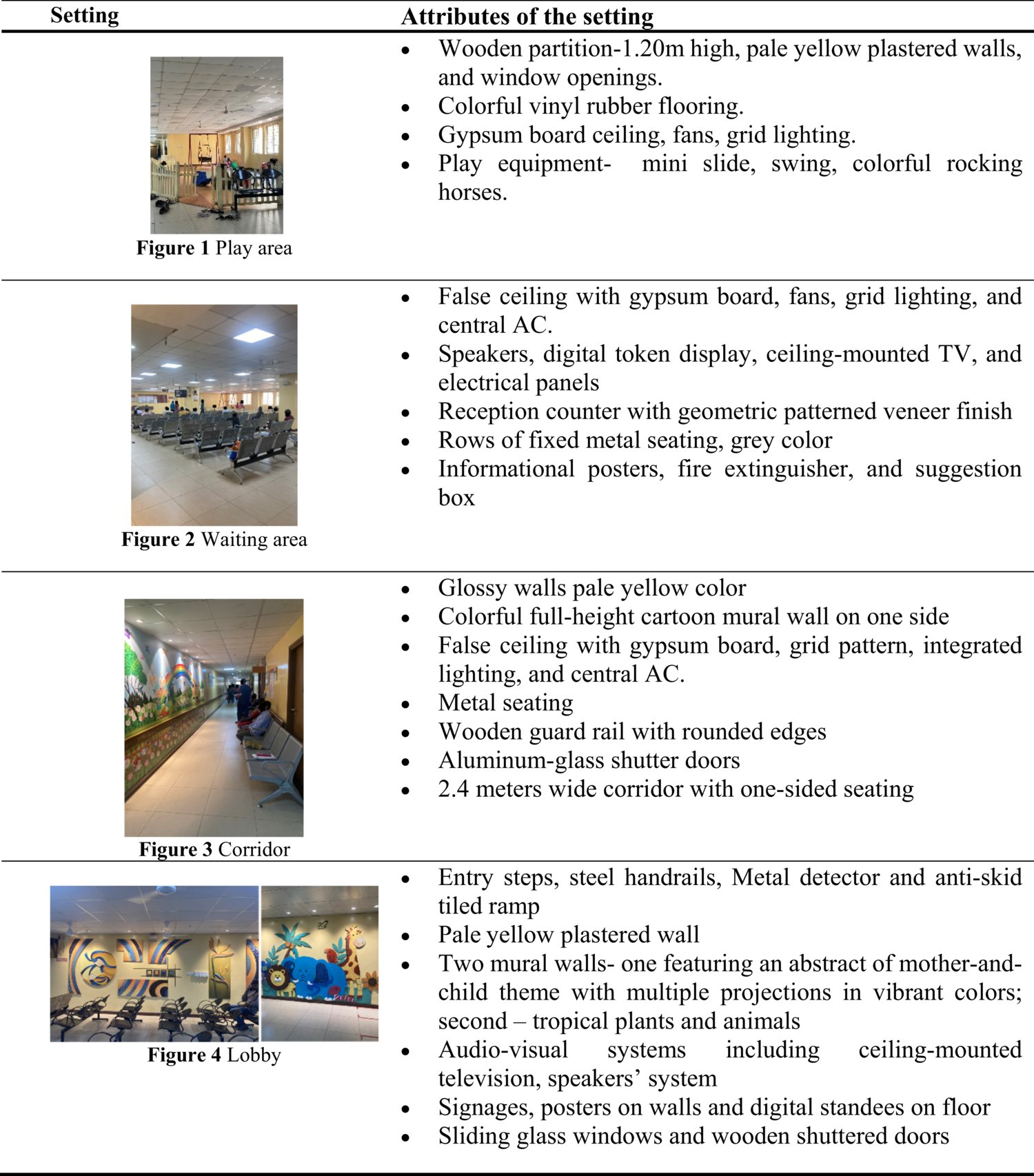

**Figure 1.**
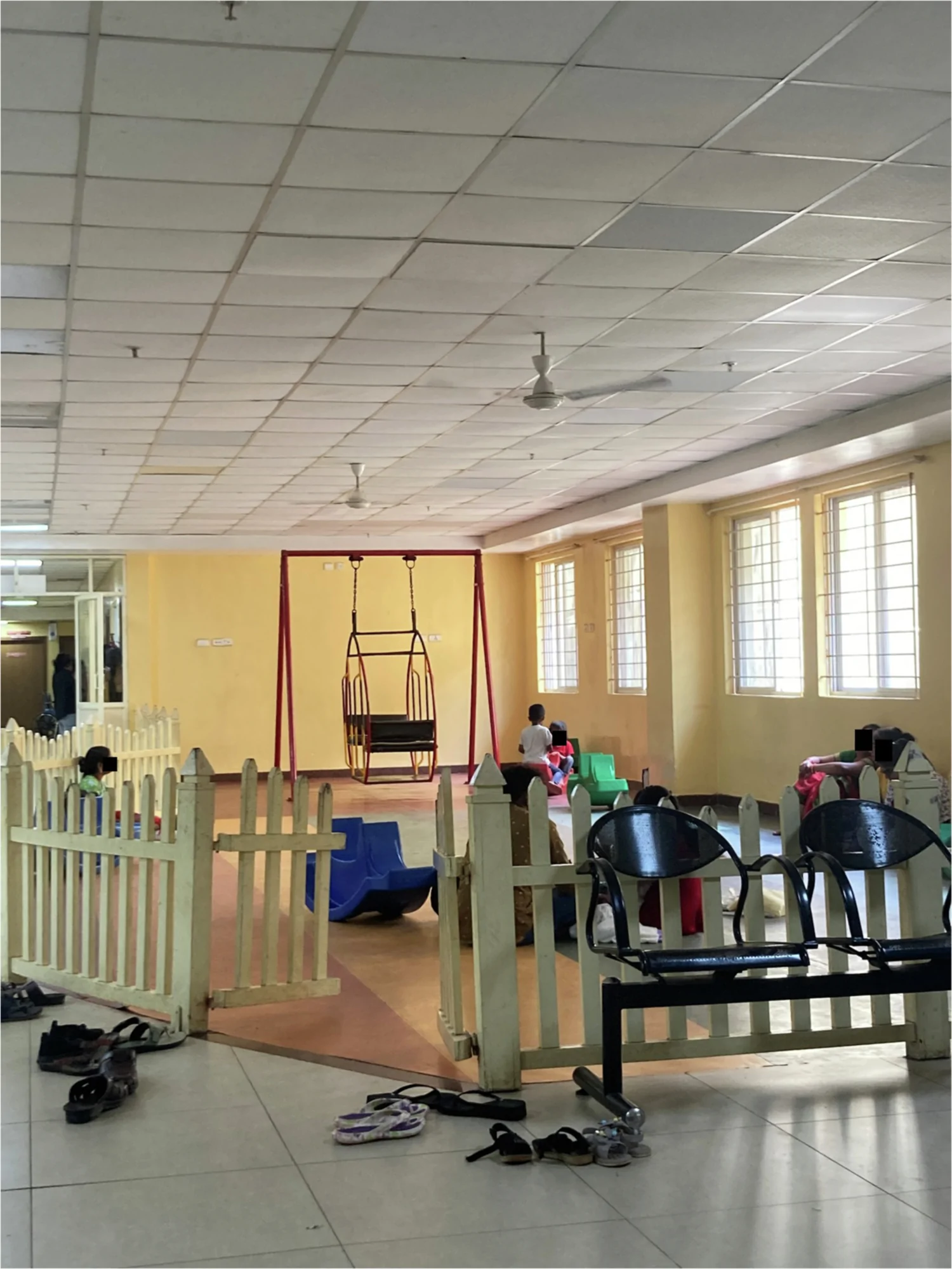
Play area.

**Figure 2.**
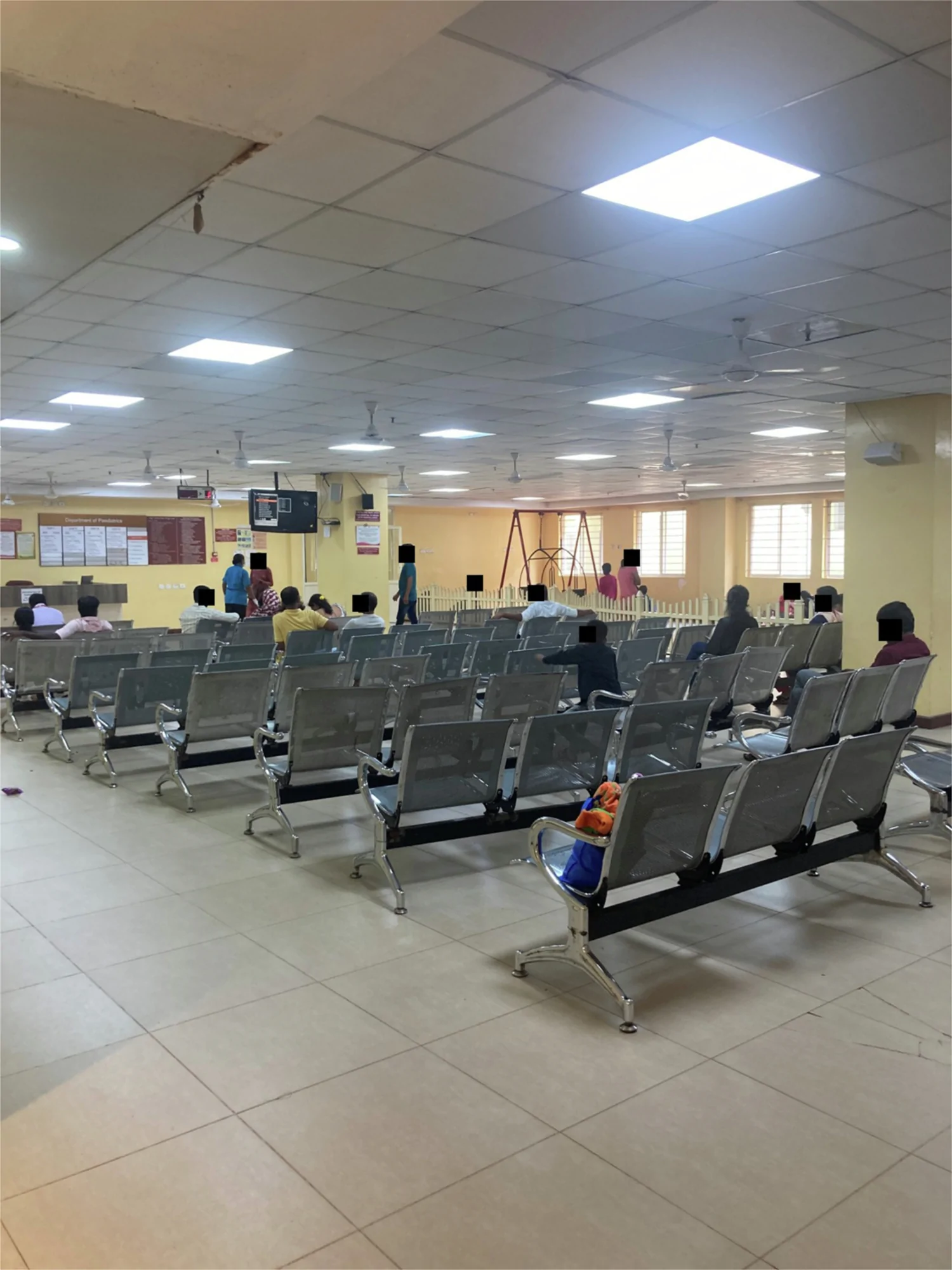
Waiting area.

**Figure 3.**
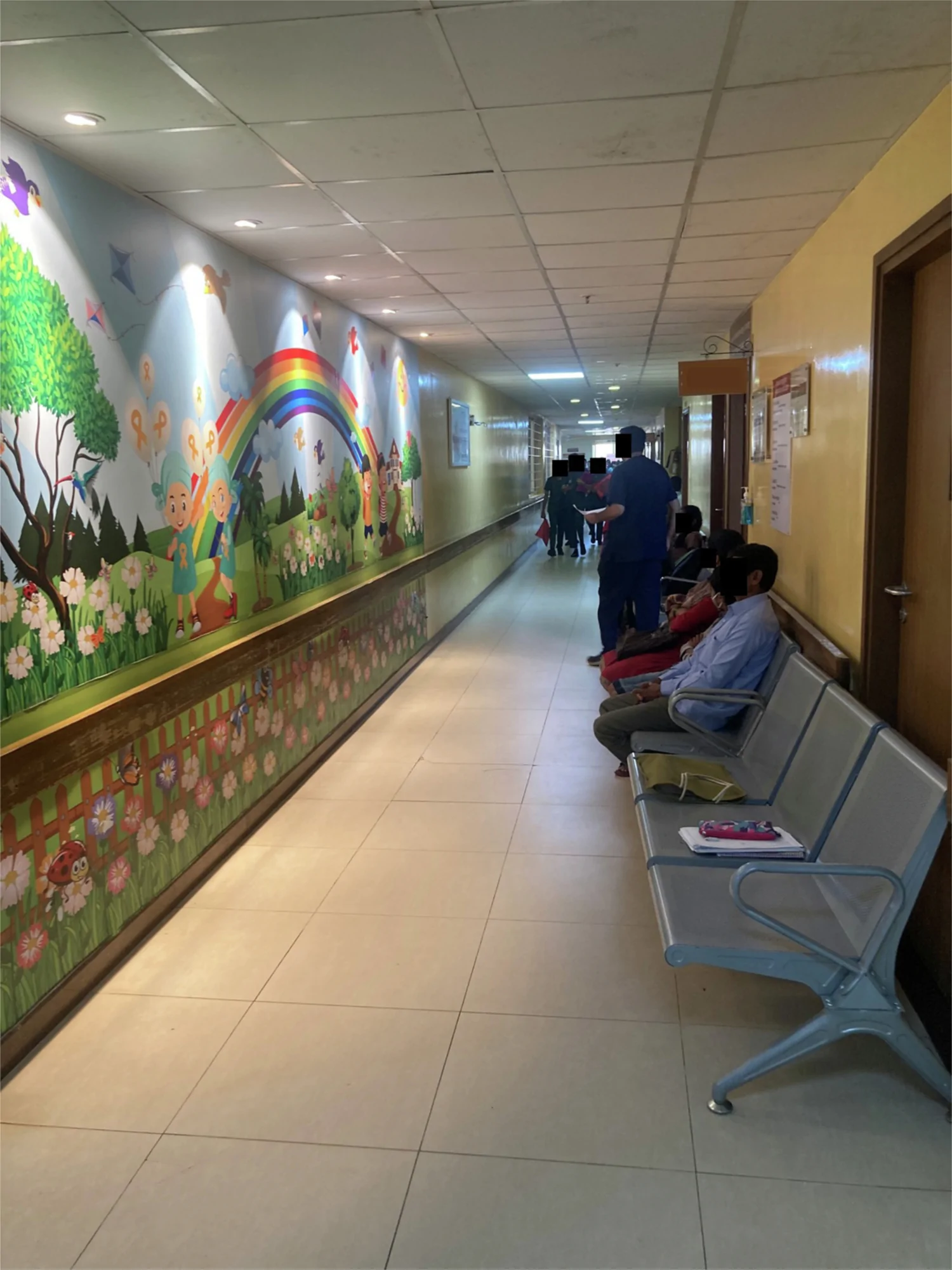
Corridor.

**Figure 4.**
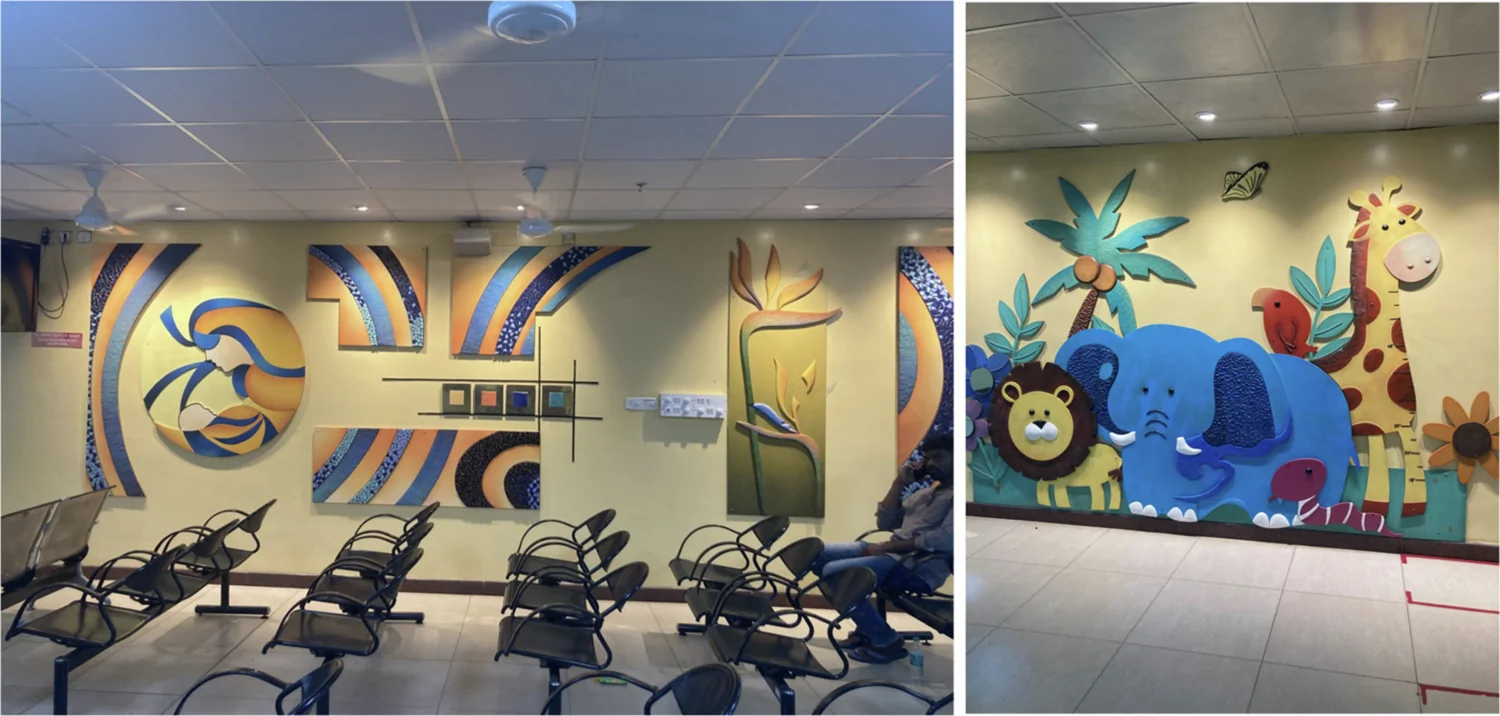
Lobby.

Figures 1–4 illustrate the four study settings included in the study. Figure 1 shows the play area with child-friendly play equipment and colourful interiors; Figure 2 depicts the waiting area with seating, reception facilities, and informational displays; Figure 3 illustrates the corridor featuring mural walls and circulation space; and Figure 4 presents the lobby characterized by thematic mural walls, signage, and visual displays. Table II summarizes the physical characteristics of these settings, which together represent a variety of everyday HPE encountered by children during healthcare visits.

Together, these settings represent a continuum of everyday hospital spaces that children routinely encounter during healthcare visits, allowing the study to examine how variations in physical environmental attributes influence children's interactions and experiences.

## 4. Materials and methods

This study adopts an environment–behaviour research design using a multi-method qualitative approach to examine how children engage with everyday HPE through their interactions, representations, and verbal interpretations of spatial features. This multi-method qualitative approach combines observations, drawings, and verbal elicitations to also support methodological triangulation across multiple complementary data sources. Perspectives from environmental psychology informed the analytical framework and helped interpret children's engagement within healthcare settings (Barrett & Twycross, 2018; Creswell, 2014). The integration of behavioural interaction patterns alongside children's visual expressions and verbal interpretations of environmental features enabled a more comprehensive understanding of how physical attributes of healthcare settings shape children's experiences within pediatric environments (Creswell, 2014).

The study is grounded in a constructivist paradigm, which views children as active interpreters of their surroundings. In line with this position, observations, drawings, and verbal elicitations were chosen because they capture complementary dimensions of children's experiences (Clark, 2008; Greig et al., 2013; Guest et al., 2013). Observation documents children's natural interactions with environmental features (Cosco et al., 2010; Greig et al., 2013; drawings offer visual representations of experiences that may be difficult to verbalize (Brooks, 2009; Clark, 2008; Drake & Winner, 2013); and verbal elicitations allow children to express the meanings associated with their behaviours and drawings (Angelöw & Psouni, 2025; Clark, 2008; Creswell, 2014; Greig et al., 2013). The sequential integration of these methods thus supports a child-centered understanding of how children construct their experiences within healthcare environments.

**Figure 5.**
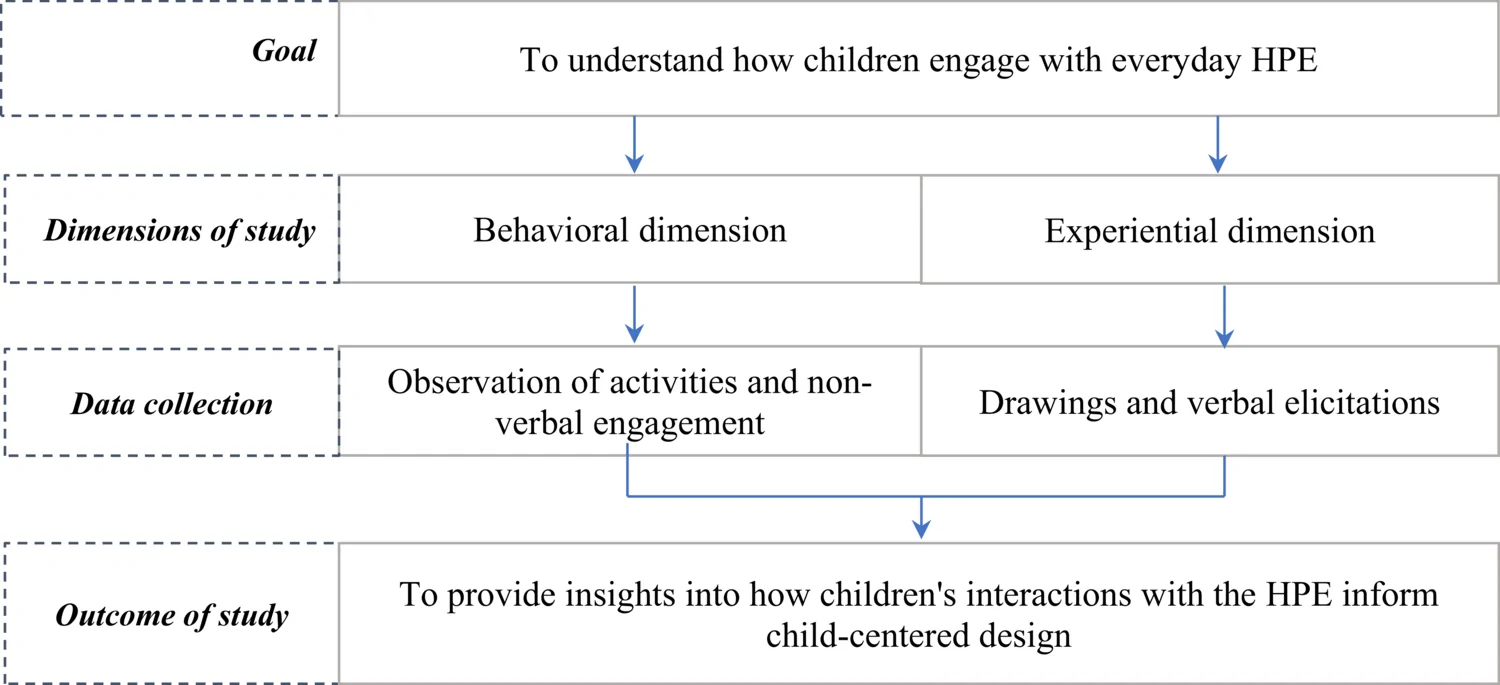
Outline of this study (source: authors).

As shown in Figure 5, the study examines children's experiences within the HPE by exploring their behaviours and perceptions of spatial attributes across non-clinical hospital settings. Observational data were analyzed through descriptive behavioural mapping to identify recurring patterns of engagement across selected settings, while children's drawings and verbal responses were thematically analyzed using ATLAS.ti software.

Data collection was conducted by the first author across the play area, waiting area, corridor, and lobby. All participants were studied within the same spatial context in which they were naturally present during their hospital visits. This ensured contextual consistency and supported a richer understanding of children's experiences within their HPE. The procedures used for participant selection and data collection are described below.

### 4.1. Participant details

A total of 25 participants out of 28 children, aged between 5 and 18 years, were included in this study. This age group was selected because children within these developmental stages are generally able to express their experiences through verbal and non-verbal methods such as drawing and conversation (Angelöw & Psouni, 2025; Greig et al., 2013) while also representing diverse stages of cognitive, social, and environmental understanding (Greig et al., 2013). Participants were recruited through convenience sampling using a direct on-site identification from four commonly used hospital spaces: the play area (*n* = 7), waiting area (*n* = 8), corridor (*n* = 5), and lobby (*n* = 5), reflecting the natural availability of children within these commonly used hospital spaces. Following earlier child-centered environmental research approaches (Greig et al., 2013), data collection was primarily conducted when children were most available during weekends or after school hours (15:30 onwards). These time periods were intentionally selected to maximize the availability of children within common hospital spaces and may have resulted in greater representation of visitors compared to children attending during school hours or weekdays. During these periods, the researcher approached children and their accompanying caregivers who were present in the selected settings and met the inclusion and exclusion criteria outlined in Table III.

**Table III.** Inclusion and exclusion criteria of study (source: authors).

Criteria	Description
Inclusion criteria	✓ Children of all genders ✓ Children aged 5–18 were included ✓ Children visiting common areas of hospital setting
Exclusion criteria	✓ Children with visible illness ✓ Children admitted to inpatient wards

Participants included children of all genders, aged between 5 and 18 years, who were present in the common areas of the hospital during the data collection period. Children with visible illnesses and those admitted to inpatient wards were excluded from participation, as their mobility and access to common areas were restricted during the data collection period. During the recruitment period, 28 children and their accompanying caregivers who met the inclusion criteria were approached for participation. Following the initial observation phase, three caregivers declined participation in the subsequent drawings and verbal elicitation activities. In accordance with the approved ethical protocol, all observation records, field notes, and photographs associated with these participants were deleted and excluded from analysis. Consequently, the final study sample comprised 25 participants. Details regarding the consent process, participant refusals, and handling of excluded observation records are described in Section 4.2.

### 4.2. Ethical considerations

Ethical approval for this study was obtained from the Institutional Ethics Committee (IEC) of Kasturba Hospital–Kasturba Medical College (KH–KMC), Manipal Academy of Higher Education (MAHE), Manipal (Approval No. IEC1:334/2022), under the category of an observational study, prior to data collection. Following IEC approval, the study was prospectively registered with the Clinical Trials Registry–India (CTRI) (Registration No. CTRI/2023/06/053554). The approved study protocol included a staged data collection procedure beginning with unobtrusive observation in the initial phase followed by drawings and verbal elicitation activities conducted after obtaining parental consent and child assent. Throughout the study, participant rights, informed consent, privacy and confidentiality, protection of vulnerable participants, and risk minimization were strictly maintained.

Research involving children requires careful consideration of both ethical protection and meaningful participation (Angelöw & Psouni, 2025; Barrett & Twycross, 2018; Creswell, 2014; Einarsdóttir, 2007; Greig et al., 2013; Hunleth et al., 2022). In the present study, the initial observation phase was designed to capture children's natural interactions with the healthcare environment. Naturalistic observation is widely employed in child-centered and environmental-behavioural research because prior awareness of being observed may influence children's behaviours (Cosco et al., 2010; Greig et al., 2013; Guest et al., 2013). Studies exploring children's experiences within non-clinical settings frequently employ unobtrusive observation to minimize behavioural reactivity while preserving contextual authenticity, while subsequent consent and assent procedures ensure ethical participation and respect for children's rights (Angelöw & Psouni, 2025; Barrett & Twycross, 2018; Creswell, 2014; Hunleth et al., 2022).

Accordingly, only children who met the inclusion criteria and were present within the selected setting were unobtrusively observed within the common areas before being approached for participation in the subsequent phase of drawings and verbal elicitation activities. During this phase of unobtrusive observation, data collection containing observation maps, field notes and photographs was restricted to only non-identifiable behavioural and environmental interactions before consent. Photographs were taken to document the physical setting, and only children's interactions with environmental features with no personal identifiers, facial images or other identifying characteristics were recorded.

Following the observation period, caregivers and children were approached and provided with participant information sheets tailored for adults and children, respectively (Angelöw & Psouni, 2025; Creswell, 2014; Einarsdóttir, 2007). Written parental consent and child assent were then obtained prior to the drawings and verbal elicitation activities. Participation was voluntary, and children were informed that they could decline or withdraw without consequence. Twenty-eight children were initially observed. Following the observation phase, three families declined participation during the second phase (drawings and verbal elicitations). Observation records, field notes, and photographs associated with those three participants were immediately deleted and excluded from the study. Thus, the final dataset therefore comprised 25 participants.

Several safeguards were implemented to protect the privacy and confidentiality of the included participants (Einarsdóttir, 2007; Hunleth et al., 2022). Only non-identifiable behavioural and environmental interactions were recorded during the observation phase, and no identifiable data was recorded until consent was obtained. Observations were conducted only within common waiting and circulation spaces and not within consultation rooms, treatment areas, or other locations involving private interactions. The researcher remained in a fixed, non-intrusive position within the setting and did not interact with participants during observation. Collected data were coded, anonymized, and securely stored on password-protected devices accessible only to the authors. All photographs used for analysis and publication were anonymized and screened to ensure that participant identities were not visible. These procedures were implemented in accordance with established ethical guidance for research involving children and observational studies in naturalistic settings (Angelöw & Psouni, 2025; Einarsdóttir, 2007; Greig et al., 2013).

### 4.3. Data collection

Data collection was conducted in the common areas of the pediatric department of a general hospital, which are considered non-clinical spaces where children naturally spend time during their hospital visits. The procedure was carried out by the first author, who positioned herself in the setting to unobtrusively document children's behavioural engagement before introducing the drawing and verbal elicitation activities. The observational phase followed the approved study protocol by the IEC and was intended to capture children's natural interactions with the healthcare environment. Only non-identifiable behavioural and environmental interactions were recorded during this stage, and no identifiable data was recorded until consent was obtained. Such naturalistic observation is widely used in child-centered and environmental behaviour research because direct engagement prior to observation may influence children's behaviours and reduce the authenticity of observed interactions (Cosco et al., 2010; Greig et al., 2013; Guest et al., 2013).

Following the observation stage, caregivers and children were approached, provided with age-appropriate participant information sheets, and invited to participate in the drawings and verbal elicitation activities within the setting, which served as a child-centred method for studying their perceptions of the environment. This approach supported the development of rapport with participants while enabling children to express their experiences through visual and verbal modes. The drawings then guided short verbal elicitation sessions in which children explained the meaning of their sketches using open-ended prompts, allowing deeper insight into how they interpreted environmental features within the HPE. Together, this sequential use of observations, drawings, and verbal elicitations enabled a contextually grounded understanding of children's engagement with everyday healthcare environments.

#### 4.3.1. Observation

The main purpose of observation is to understand how children naturally interact with their surrounding environment, how they navigate space, and what environmental features they use or avoid (Cosco et al., 2010). According to Cosco et al. (2010) and Guest et al. (2013), observation yields insights into children's interactions and behaviours in the physical setting. The observations focused on non-identifiable behaviours such as movement patterns, posture, engagement with objects in the settings, and verbal/non-verbal behavioural cues as derived from prior environmental-behaviour studies (Barrett & Twycross, 2018; Guest et al., 2013; Lambert et al., 2014; Ulrich, 1991). No personal identifiers were recorded during this phase. Children's behaviours and their interactions were documented using annotated observation maps (Supplementary information-2, SI-2), template of behavioural mapping of children's behaviours (Supplementary information-4, SI-4) and field notes. The templates for observation maps and behavioural mapping were prepared based on the literature sources, reviewed by both authors, and pilot tested in two different settings. Observation has been widely employed in child-environment and educational research to understand children's behaviours, environmental interactions, and use of space through direct observation and behaviour mapping approaches (Cosco et al., 2010; Ferguson et al., 2013). However, within healthcare research, studies have more commonly relied on interviews, surveys, and caregiver reports, with comparatively fewer investigations employing direct observation to examine how children naturally engage with healthcare environments (Einarsdóttir, 2007; Melles et al., 2020; Sevón et al., 2023; Williams et al., 2022). Given the study's aim to understand children's interactions with environmental features within everyday healthcare settings, observation was considered an appropriate method. The observation process was conducted in two stages, as outlined below:

(A) Stage 1: observing the setting

The four hospital settings were documented using the modified setting framework inspired by Rapoport (1990) presented in Table I. Basic measuring tools captured the spatial information of the physical settings. The setting was then sketched, photographed and subsequently drafted using architectural software, AutoCAD, to prepare setting layouts required for observing children's interaction in the same setting. For reference, the sample of an observation map is provided in supplementary information-2 (SI-2).

(B) Stage 2: observing the participant in the setting

Participants in the healthcare settings were observed unobtrusively through behaviour mapping. This method is one of the few tools that allow researchers to study and document both behaviour and environmental context (Cosco et al., 2010). The lead researcher positioned herself in a static corner to maintain anonymity and minimize disruption while also ensuring a wide-angle view (Cosco et al., 2010). Observations were conducted across four everyday healthcare settings – play area, waiting area, corridor, and lobby. Though a total of 28 children were initially observed, the final dataset included 25 participants after the decline in participation of three participants.

Given the dynamic nature of children and their tendency to move in and out of the setting, a 30-min observation period was considered appropriate to capture a range of naturally occurring interactions, movement patterns, and engagement with environmental features while maintaining consistency across participants (Barrett & Twycross, 2018; Cosco et al., 2010; Guest et al., 2013). Behavioural categories such as playing, observing, jumping, touching, standing, climbing, and sitting were operationally defined prior to data collection based on established behaviour-mapping and observational approaches in child-environment and pediatric environmental behaviour research (Cosco et al., 2010; Guest et al., 2013). Behaviours were not treated as mutually exclusive, as participants frequently transitioned between different actions within the observation period. Participant's behaviours were observed continuously for a single 30-min period within the setting where they were naturally present. Thus, a cumulative observation duration of approximately 750 min conducted across varied days and times. Supplementary information (SI-4) includes a sample behavioural mapping template which was used to document children's behaviours within the setting.

#### 4.3.2. Drawings

Drawing provides children with an opportunity to communicate experiences, perceptions and complex ideas by visually representing their thoughts on canvas that may not be easily expressed verbally (Brooks, 2009; Drake & Winner, 2013). This method was conducted with the same participants and within the same physical setting in which observations took place, thereby maintaining continuity between the observed behaviours and children's subsequent representations of the environment. Following the observation phase, caregivers and children were approached for participation, and the drawing activity began after the permission with the same child who was initially observed for 30 min. This activity lasted for about 10–20 min depending on the child's age, level of engagement and pace of participation. Twenty-five participants completed the drawing activity.

Each participant was provided with a paper (210 × 297 mm) and a 12-colour set of crayons to draw and colour. A few context-specific open-ended prompts were asked verbally to guide children during the drawing activity (Angelöw & Psouni, 2025; Brooks, 2009; Greig et al., 2013). Some of the prompts used during the activity are outlined below:

Prompt 1: “*Would you like to draw something that you like in this place?”*
Prompt 2: *“What is your favourite corner/object in this place?”*
Prompt 3: “*Is there anything that you do not like here?"*

After completing their drawings, children were invited to explain their sketch verbally. These verbal elicitations were audio-recorded with consent or documented as field notes when recording was declined. The drawings were collected and later coded using a structured coding scheme for the analysis phase.

#### 4.3.3. Verbal elicitations

Verbal elicitations were conducted immediately after the drawing session with the same participants and in the same setting. Each elicitation lasted approximately 10–20 min, depending on the child's attention span, comfort level, and cognitive abilities (Spencer & Blades, 2006). The prompts used are provided in Supplementary Information-3 (SI-3). Five participants preferred to communicate in the regional language, Kannada, while the remaining used the English language. Their responses were audio-recorded using a voice recorder app in iPhone SE (2020 model). The Kannada audio recordings were first transcribed verbatim in the original language by a bilingual researcher proficient in both Kannada and English. The transcripts were subsequently translated into English for thematic analysis prior to coding. To maintain accuracy and contextual meaning, the translated data was looked at alongside original transcripts throughout the analysis process. For participants who did not provide consent for audio recording, field notes were taken on-site and subsequently included in the coding and analysis.

The total duration for the three methods resulted in 50–70 min per participant. Observation lasted 30 min, followed by drawing (10–20 min) and verbal elicitation (10–20 min), resulting in an overall interaction time of a maximum of 70 min for each participant in their respective setting. The sequencing of these three methods within the same setting enabled a continuous and contextual understanding of children's experiences.

#### 4.3.4. Strength of chosen methods

The main strength of the study is the integration of three methods in a single study which were applied to each participant within the same context. These three methods were articulated in sequence that allowed each phase to build upon the other, providing an understanding of how children interacted and perceived the HPE. Combined analysis of behavioural, visual and verbal data was performed to get a comprehensive understanding of children's experiences (Creswell, 2014; O'Cathain et al., 2010).

This approach improved the study's internal validity more robustly. Firstly, triangulation was enabled due to the comparison of the findings of the methods. The results compared with what children did (observations), what they drew (drawings), and what they expressed (elicitations). Secondly, contextual uniformity – performing all the methods in the same context where the child was first seen – made the environment more consistent in their drawings, and their experiences were directly related to the physical environment that was observed.

### 4.4. Data analysis

The dataset for this study consisted of three complementary sources: (a) field notes and observation maps generated through unobtrusive behavioural observation, (b) children's drawings produced during the art-based activity, and (c) verbatim transcriptions of verbal elicitation sessions during the drawing activity. Observational data were descriptively analyzed to identify recurring patterns of behavioural engagement across settings, while drawings and verbal elicitation's data were analyzed thematically. Children's drawings and verbal data were analyzed thematically, and observational data were descriptively analyzed to identify patterns of behavioural engagement across settings. The analysis followed an inductive approach, allowing themes to emerge from the data. Supportive design theory informed the subsequent interpretation of the themes and was used to examine how environmental features identified through behavioural observations, drawings, and verbal elicitations may relate to positive distraction, engagement, and coping within healthcare environments. The integration of these datasets enabled triangulation of findings and promoted a comprehensive understanding of children's interactions within everyday healthcare settings (O'Cathain et al., 2010).

#### 4.4.1. Analysis of observational data

The observational dataset consisted of observation maps and field notes capturing children's behaviours within the physical environment. Behavioural categories recorded for children included playing, observing, jumping, touching, standing, climbing, and sitting. Each discrete occurrence of a behaviour by a participant was recorded as one frequency count during the 30-min observation period. One participant contributed multiple counts to the same behavioural category if the behaviour occurred repeatedly during the observation period. Behavioural occurrences were recorded in real time on observation maps and field notes and later reviewed during analysis. Adapted from Cosco et al. (2010), this descriptive behavioural mapping approach was used to identify recurring patterns of engagement, enabling an in-depth analysis of children's interactions with the physical across hospital settings.

#### 4.4.2. Analysis of drawings and verbal elicitations

Children's drawings were analyzed in two stages. First, each drawing was systematically reviewed and coded for visual characteristics such as objects, colours, spatial features, activities and symbols represented within the drawing. This stage provided a systematic inventory of the visual elements depicted by the children. Second, the drawings were analyzed in line with corresponding verbal elicitation responses and field notes. The participant's explanation served as a primary basis for interpreting the meanings associated with the elements in the drawing, thereby reducing reliance on researcher assumptions. Visual coding categories were developed inductively through repeated examination of the drawings and their accompanying narratives and field notes. Similar visual codes and meanings were grouped into broader categories. For instance, individual visual elements represented in the drawings, such as swings, slides, aquariums, television screens, clocks, ceiling lights, trees, mural graphics, balloons, wall posters, signboards and seating areas, were initially identified and coded. Related visual codes and meanings were grouped into broader categories: play and activity features, nature or biophilic elements, digital and interactive features, visual and decorative features, sensory features, furniture and seating elements, functional and safety features, educational elements, and symbolic or imaginative representations. These categories were further refined through comparison with participants' verbal elicitations and thematic analysis of the elicitation data.

The transcribed verbal data were analyzed using thematic analysis framework proposed by Braun and Clarke (2006) together with the drawings. The analysis was conducted by the first author using ATLAS.ti and was periodically reviewed by the second author. Firstly, audio recordings were listened to repeatedly to capture children's tone and emotional expressions, and transcripts were re-read to facilitate line-by-line coding. Coding focused on identifying segments, words, or phrases related to the study objective, i.e., behavioural and experiential responses to the physical environment. Initial codes generated from the verbal data were compared with visual codes derived from the drawings, and related codes were subsequently organized into broader categories and themes proposed by Braun and Clarke (2006). To enhance analytical rigour and coding consistency, the coding framework, code definitions, category development, and emerging themes were continually reviewed and discussed with the second author throughout the interpretive process. Any differences in interpretation were resolved through iterative discussion and re-reviewing of the original observation records, drawings, verbal elicitation transcripts, and field notes until consensus was reached. Recurring themes were traced across all participants and subsequently refined to ensure they remained coherent, representative, and meaningfully aligned with the focus of study. Through this reflexive approach, three overarching themes and several related sub-themes were constructed (Table VII), capturing how children interacted with environmental attributes within everyday HPE.

Overall, the analysis followed a sequential and interpretive process. Behavioural mapping served as the initial step, identifying patterns of interaction among participants within the physical environment. This was followed by children's drawing to identify key environmental elements; alongside verbal elicitation transcripts were analyzed to understand the meanings and preferences associated with these elements. In the final stage, the behavioural, visual, and verbal datasets were triangulated to identify convergent patterns and generate themes capturing children's experiences within the healthcare environments.

### 4.5. Qualitative rigor and trustworthiness

Qualitative rigour was maintained in this study by addressing the criteria of credibility, transferability, dependability, confirmability and reflexivity (Braun & Clarke, 2006). The Standards for Reporting Qualitative Research (SRQR) framework guided the study design, data collection procedures, and analytical process to support methodological transparency (O'Brien et al., 2014). Credibility was strengthened through methodological triangulation comparing recurring patterns of children's engagement across behavioural observations, drawings and verbal elicitations. This cross-validation allowed children's verbal data to offer contextual support for the observed behaviours and drawings (Braun & Clarke, 2006; Denzin, 1978). Transferability was addressed through detailed descriptions of study settings, participant characteristics, and environmental attributes within the HPE, allowing readers to assess the applicability of findings that may be applicable to similar contexts (O'Brien et al., 2014). Dependability was ensured through systematic observation protocols and consistent coding procedures applied across all the settings and supported a transparent and iterative analytical process (Braun & Clarke, 2006). Confirmability was strengthened by presenting illustrative participant excerpts alongside behavioural evidence, ensuring the interpretations remained firmly grounded in the data and were supported across multiple sources (Braun & Clarke, 2006).

Reflexivity was maintained throughout the research process by acknowledging the researcher's interpretive role during data collection and analysis (Braun & Clarke, 2006). To minimize bias, findings were derived through triangulation of behavioural observations, drawings, and verbal elicitation data, with participants' verbal elicitations offering contextual support for interpreting visual representations. Coding and theme development followed an iterative process, with analytical decisions periodically reviewed and discussed with the second author. These procedures supported data-grounded interpretations and enhanced the trustworthiness of the findings (Braun & Clarke, 2006; O'Brien et al., 2014).

### 4.6. Researcher positionality and reflexivity

The study was led by the first author, an architect and researcher with experience in environment–behaviour research, and carried out under the guidance of the second author. The researchers' background in architectural design and spatial analysis guided the interpretation of children's interaction with healthcare environments. At the same time, reflexive awareness was maintained to minimize potential bias in observing and interpreting children's behaviours and expressions. To minimize interference by the researcher, data were collected in naturalistic settings and interpreted through triangulation of observations, drawings, and verbal elicitation. The use of children's direct verbal elicitations alongside visual data further helped ensure that findings reflected participants' perspectives rather than researcher assumptions.

## 5. Results

The findings of the research revealed children's perceptions and behaviours across four different hospital locations. Three datasets were generated through a multi-method qualitative approach to understand children's behaviours and experiences within the HPE. The thematic analysis of drawings and verbal elicitations identified three prevalent themes: (1) *Familiar environmental engagement*, where children interacted with recognizable environmental features that supported comfort and attention; (2) *Curiosity-driven environmental engagement*, reflecting responses to novel sensory elements that encouraged exploration; and (3) *Inspirational environmental engagement*, where children related everyday functional features to imagination and learning during their hospital visits. These themes represented distinct ways in which the physical environment shaped children's behavioural and experiential engagement within the HPE.

### 5.1. Participant profile

The study included 25 participants recruited through a convenience sampling approach across four healthcare settings. The sample comprised 64% female (*n* = 16) and 36% male (*n* = 9) participants. Participants ranged from 5 to 18 years and were grouped into five developmental age categories: 5–7 years (28%), 8–10 years (36%), 11–13 years (16%), 14–16 years (12%), and 17–18 years (8%), with the highest representation in the 8–10-year age group and the lowest representation in the 17–18-year group. These age categories were grounded in developmental psychology and childhood research literature, which suggest that children undergo progressive changes in cognitive reasoning, communication abilities, environmental understanding, and modes of participation from early childhood through adolescence (Angelöw & Psouni, 2025; Greig et al., 2013; Piaget, 1950; Spencer & Blades, 2006).

Table IV presents the participant profile linking age, gender, and observed setting. Although there was an unequal gender distribution, the analysis focused on overall behavioural and perceptual patterns rather than gender-based comparisons, thereby minimizing any potential bias in the interpretation.

**Table IV.** Participant's age group intervals and gender distribution.

Participant ID	Age (in years)	Gender	Study setting
P-01	9	Female	Play area
P-02	11	Male	Corridor
P-03	8	Female	Play area
P-04	10	Female	Lobby
P-05	9	Male	Lobby
P-06	9	Female	Waiting area
P-07	7	Female	Waiting area
P-08	6	Female	Waiting area
P-09	12	Male	Lobby
P-10	11	Female	Corridor
P-11	13	Female	Corridor
P-12	6	Male	Waiting area
P-13	8	Female	Play area
P-14	7	Female	Waiting area
P-15	9	Female	Play area
P-16	10	Male	Lobby
P-17	8	Male	Lobby
P-18	7	Female	Waiting area
P-19	10	Male	Play area
P-20	14	Female	Corridor
P-21	15	Male	Corridor
P-22	17	Female	Waiting area
P-23	8	Female	Play area
P-24	10	Female	Play area
P-25	18	Female	Waiting area

The sample size was guided by the concept of information power proposed by Malterud et al. (2016), which suggests that the more relevant information a sample provides in relation to the study aim, the fewer participants are required. The focused study aim, specificity of the participant group, and the rich multi-method qualitative approach generated data through observations, drawings, and verbal elicitations, which contributed to the adequacy of the sample. Throughout analysis, codes and categories were developed iteratively and compared across participants and data sources. During the later stages of analysis, newly collected data primarily confirmed existing codes and themes rather than generating substantially new categories. During the iterative coding process, new codes became progressively less frequent. For instance, analysis of participants 19–22 generated only a limited number of additional codes, while no substantially new codes emerged across the observations, drawings, and verbal elicitations datasets from participants 23–25. Thus, this recurrence of behavioural, visual, and verbal patterns across participants and settings indicated that sufficient information power had been achieved (Braun & Clarke, 2006; Malterud et al., 2016).

### 5.2. Findings from observation

Observation data captured both the physical attributes of the four settings and the behaviours exhibited by children within them, enabling a direct comparison between environmental configuration and behavioural response. Table V summarizes the distribution of environmental attributes across the four HPE play areas – waiting area, corridor, and lobby – categorised as Enclosures (E), Fenestrations (F), Accessories (A). The classification facilitates a structured understanding of how spatial characteristics of each setting potentially influence children's interactions within the HPE.

**Table V.** Distribution of enclosures (E), fenestrations (F), accessories (A) across the four healthcare settings (source: authors).

Settings	Enclosures	Fenestrations	Accessories
Play area	Wooden partition wall, plastered wall	Window	Play toys, swing, poster, ceiling fans, clock
Waiting area	Mural wall	Window	Reception counter, chairs, posters, television, speakers
Corridor	Plastered wall	Consultation room doors, duct door	Digital display boards, handrail, spotlights, ceiling lights
Lobby	Mural wall Glass wall	Glass door	Information desk, digital display standee, metal detector, television, reception counter

The distribution of environmental attributes across the settings illustrated how each setting had varied degrees of enclosures, fenestrations and accessory elements. The play area and lobby highlighted a higher concentration of accessories, indicating a greater presence of visually and physically engaging components; in contrast, the waiting area and corridor showed moderately limited features. Fenestrations such as windows and doors were present across all settings, supporting visual connectivity and movement, while enclosure types differed from wooden partitions and plastered walls to mural and glass walls, creating distinct settings. These variations characterized distinct spatial affordances that influenced children's engagement responses across settings.

#### 5.2.1. Descriptive behavioural mapping across settings

Behavioural mapping was used to descriptively document recurring patterns of children's behaviours and actions within each setting. Behaviours were recorded as discrete occurrences associated with locations and documented as frequency counts linked to specific environmental features (Cosco et al., 2010).

The observation data were analyzed through the frequency of occurrence counts of observed behaviours such as playing, observing, jumping, touching, climbing, and sitting- recorded in each location (Table VI). The actions reflected both active (playing, jumping, climbing, touching) and passive (observing, sitting, standing) modes of engagement within the HPE. Supplementary information-4 (SI–4) presents the template of behavioural mapping of children's behaviours.

**Table VI.** Descriptive occurrence counts of observed behaviours across four settings (source: authors).

Setting	Playing	Observing	Jumping	Touching	Standing	Climbing	Sitting	Total counts
	(P)	(O)	(J)	(T)	(St)	(C)	(Stt)	
Play area	28	20	8	5	3	8	7	79
Waiting	10	21	0	3	0	0	10	44
Corridor	0	9	0	3	0	0	13	25
Lobby	10	14	3	5	15	6	8	61
Total counts	48	64	11	16	18	14	38	

Note: counts represent discrete behavioural occurrences recorded during the observation period and are presented descriptively rather than for statistical comparison.

Behavioural mapping identified distinct patterns of engagement across the four healthcare settings, corresponding to the environmental characteristics. The play area was categorized by a wide range of active behaviours, including playing (28), jumping (8), and climbing (8). The presence of interactive play equipment, colourful surfaces, and semi-enclosed partitions supported multi-sensory exploration and physical engagement, resulting in diverse interactions.

In contrast, the waiting area was primarily associated with observing (21) and sitting (10) behaviours, with no occurrences of jumping or climbing. Fixed seating and screen-based elements seemed to support visual engagement while limiting opportunities for active interaction.

Similarly, the corridor was characterized by sitting (13), observing (9), and occasional touching (3). Although mural elements and screen-based accessories attracted attention, its linear configuration appeared to support relatively limited interaction.

The lobby exhibited a broader range of behaviours, including standing (15), observing (14), and occasional climbing (6), touching (5). The open layout, together with the presence of digital display standees and a television, contributed to exploration despite the absence of dedicated play equipment.

Overall, the behavioural mapping illustrated that enclosures, fenestrations, and accessories appeared to support children's behavioural responses, shaping their active and passive behaviours within the HPE.

Consistent with the qualitative interpretive design of the study, these descriptive behavioural patterns were subsequently triangulated with children's drawings and verbal responses. Building on this, the data were analyzed to interpret how specific environmental elements were perceived, interpreted, and experienced by the participants. This sequential integration informed the identification of the three overarching forms of environmental engagement – familiar, curiosity-driven, and inspirational forms of engagement presented in the following sections.

### 5.3. Findings from thematic analysis

This section reports the findings from the thematic analysis of transcribed verbal data alongside drawings using the thematic analysis framework proposed by Braun and Clarke (2006). Themes were developed through an iterative process to identify recurring patterns in children's engagement with environmental features across settings. Three main themes and related sub-themes were identified (Table VII), describing how children engaged with environmental features across everyday HPE. These themes captured recurring visual, tactile, and sensory interaction patterns identified across observations, drawings and verbal elicitations.

**Table VII.** Themes and sub-themes.

Themes	Sub-themes
Familiar environmental engagement	Interaction with familiar tactile elements
	Interaction with familiar visual elements
Curiosity-driven environmental engagement	Engagement with sensory environmental features
	Exploration prompting peer interaction opportunities
Inspirational environmental engagement	Learning oriented engagement
	Meaning making beyond clinical context

The themes were subsequently interpreted through the lens of supportive design theory. Familiar environmental engagement was reflected across participants across all four settings, whereas curiosity-driven engagement emerged primarily within the lobby and inspirational engagement was observed among a smaller group of participants who associated features with learning and future aspirations. Together, these themes explain how environmental elements supported children's attention, exploration, and interpretation of healthcare spaces during their hospital visits.

#### 5.3.1. Familiar environmental engagement

Familiar environmental engagement refers to children's interaction with environmental elements that are recognisable or visually relatable, thereby supporting exploratory interactions and sustained attention within the HPE. Across the play area, waiting area, corridor, and lobby, mural graphics, play equipment, partitions, and digital display elements functioned as familiar references that children could easily interpret and relate to during their hospital visits. This theme had two sub-themes- interaction with familiar tactile and interaction with familiar visual elements.

##### 5.3.1.1. Interaction with familiar tactile elements.

Children's interactions with familiar tactile elements emerged as a recurring pattern across the observed healthcare settings. Features that could be physically touched, grasped or climbed encouraged repeated tactile exploration. In the play area, children consistently interacted with swings, slides, horsie toys, and wooden railings. Behavioural mapping illustrated that many children repeatedly climbed on the wooden railings, which were subsequently represented in their drawings and described during verbal elicitation. For example, participant P-01 described the wooden railing as resembling “…*the gate of my house*” (see Table VIII), highlighting the role of familiar spatial references in supporting recognition.

**Table VIII.** Synthesis of children's engagement with environmental features across four healthcare settings (source: authors' field observations, children's drawings and verbal elicitations).

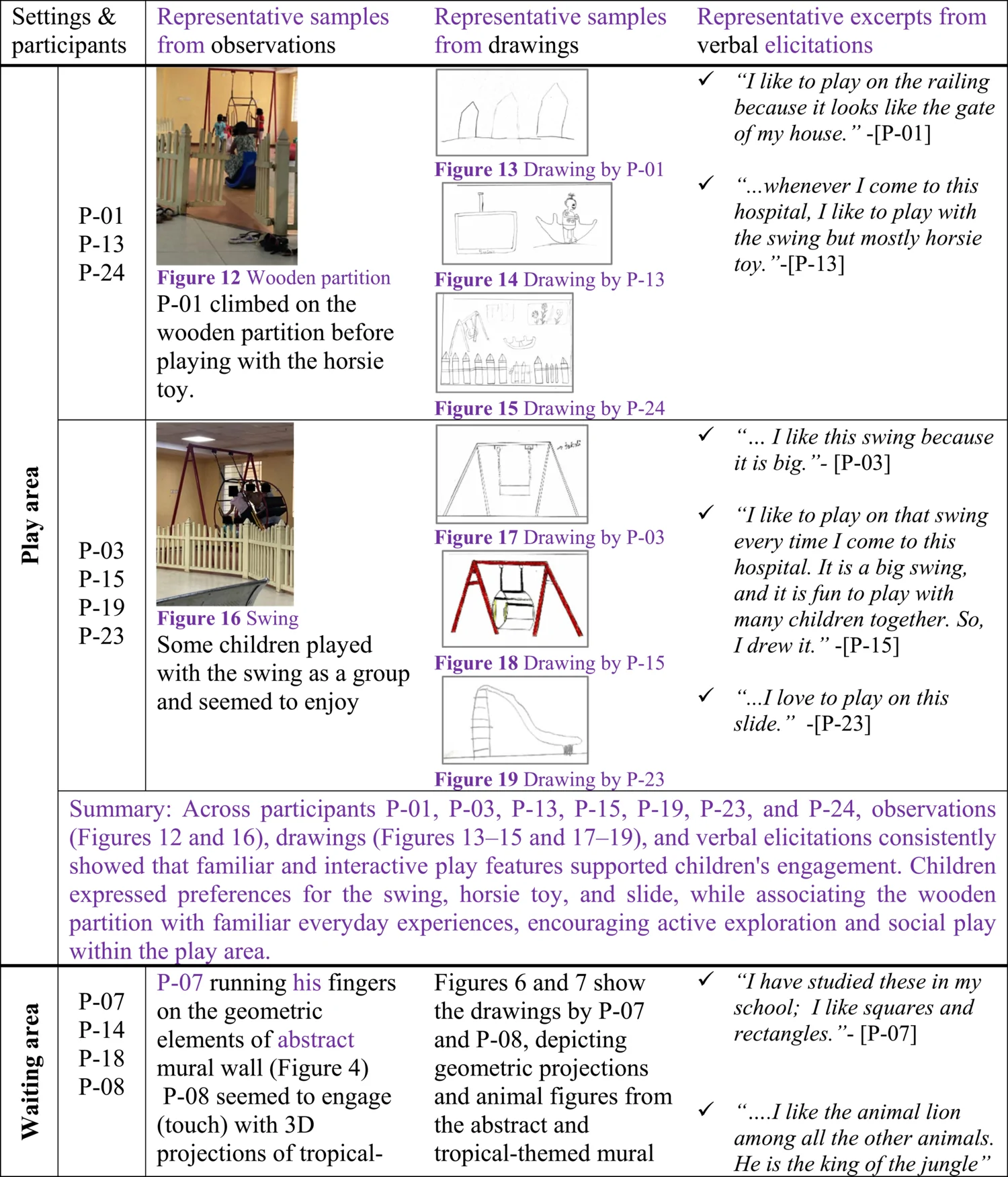 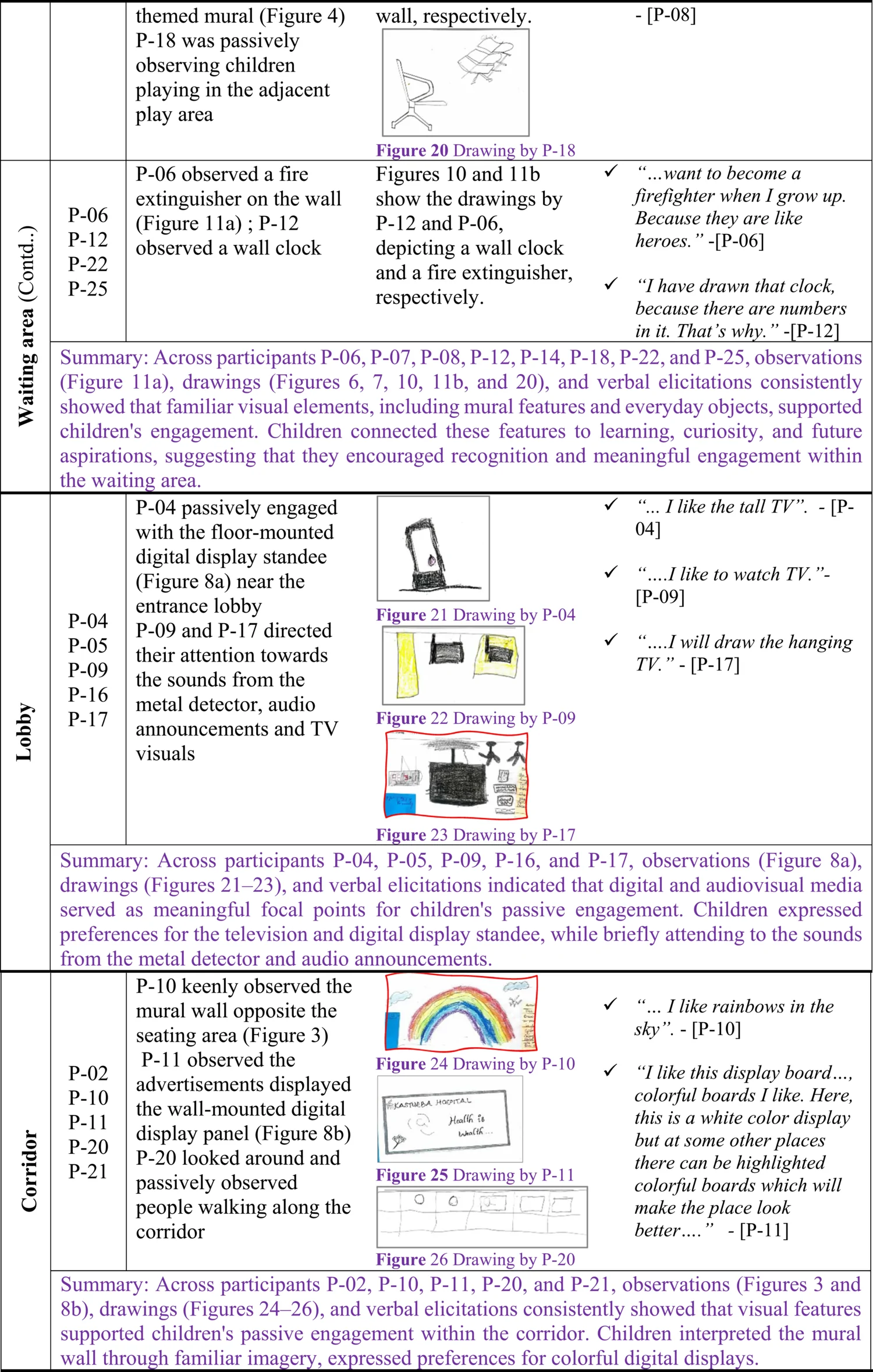

Note: To maintain readability, this table presents representative examples from observation field notes, drawings, and verbal elicitations.

✓ *"...whenever I come to this hospital, I like to play with the swing but mostly horsie toy.”* [P-13]
✓ *"..I like to play on that swing every time I come to this hospital. It is a big swing, and it is fun to play with many children together. So, I drew it.”* [P-15]
✓ *"...I love to play on this slide.”* [P-23]

Similarly, participant P-13 engaged with the horsie toy, whereas participants P-15 and P-23 preferred the swing and slide. These features were subsequently represented in their drawings (Table VIII) and enjoyable places for individual and shared play within the HPE. Collectively, the findings suggest that familiar play features encouraged active exploration and a sense of familiarity while also supporting social interaction among children during repeated hospital visits.

Across the waiting area and corridor, children frequently interacted with mural walls containing geometric patterns, rainbow elements, and animal figures. Behavioural observations showed repeated touching, tracing, and visual exploration of these features, particularly those positioned at children's eye level and within easy reach. Those elements were represented in their drawings and elaborated during verbal elicitation sessions, suggesting that recognizable mural elements supported both tactile exploration within the HPE.

##### 5.3.1.2. Interaction with familiar visual elements.

**Figure 6.**
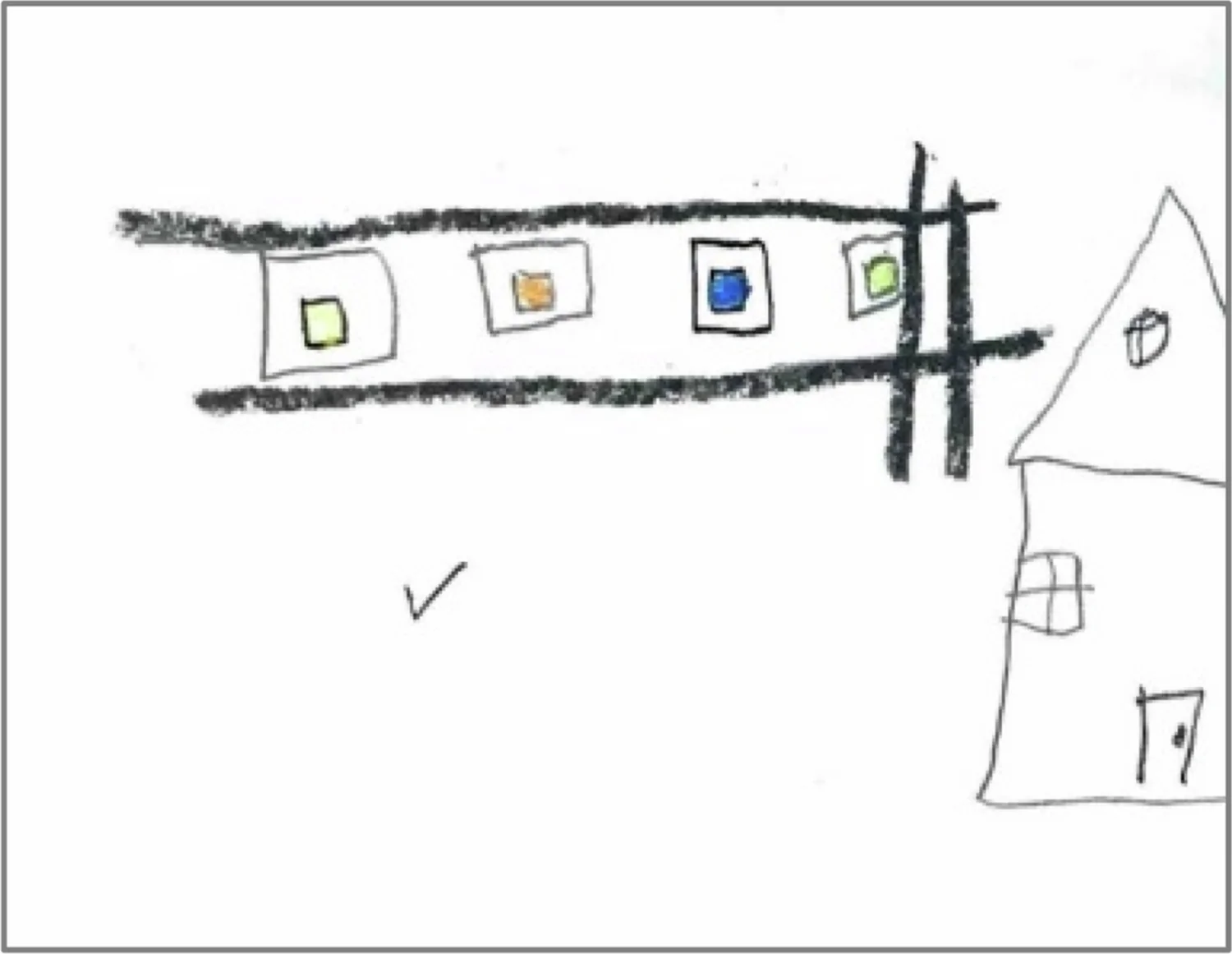
Drawing squares from the abstract mural by P-07 (modifications to drawing: none).

**Figure 7.**
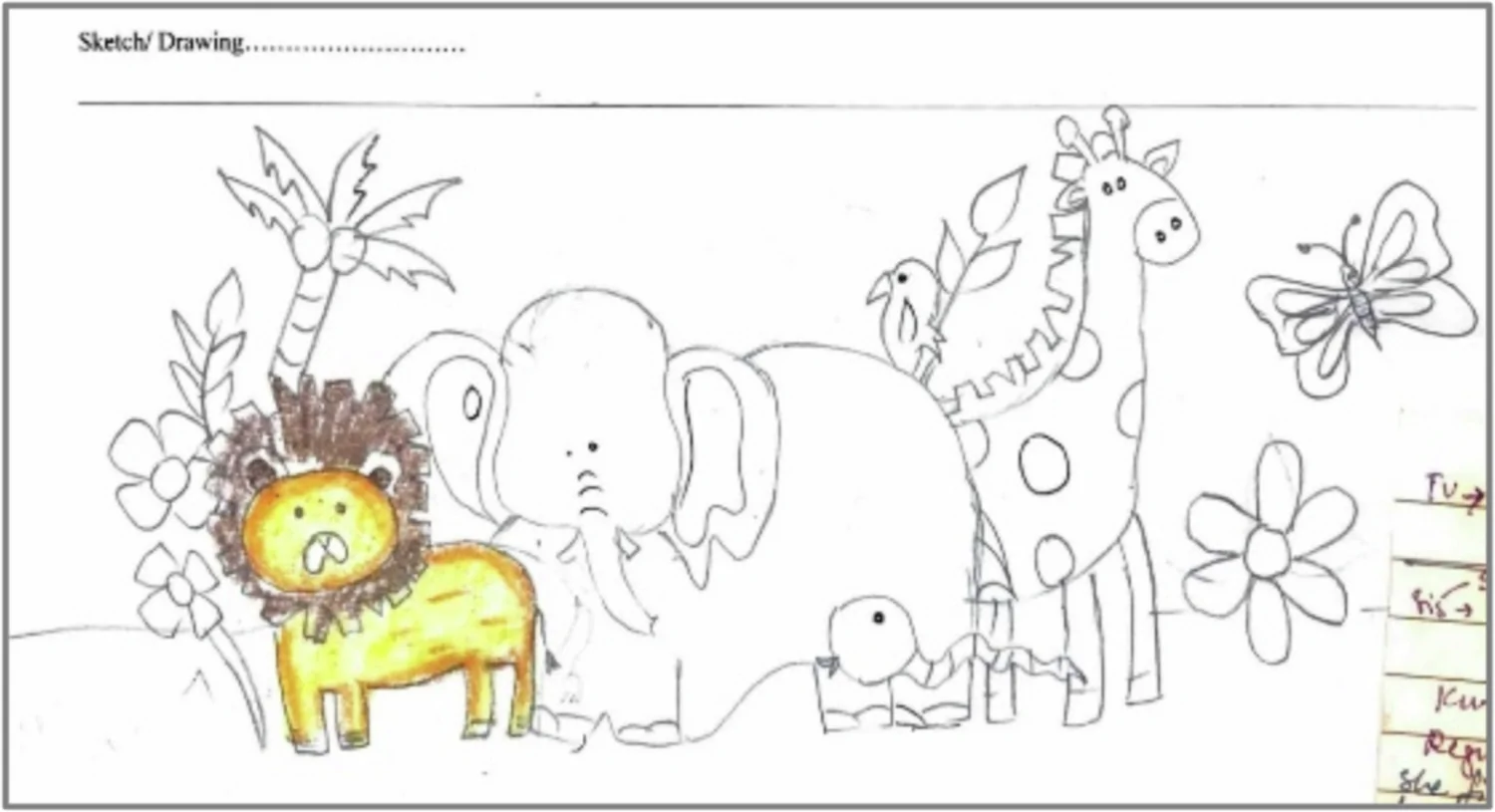
Drawing animal figures from tropical-themed mural by P-08 (modifications to drawing: none).

Children's interactions with familiar visual elements emerged as another recurring pattern across the HPE. Features that were commonly encountered in their everyday environments appeared to attract children's attention and facilitate recognition. Although the mural walls (Figure 4) featured abstract and tropical-themed compositions, children did not engage with the murals as complete artworks. Instead, they identified specific elements that attracted their attention and resonated with their individual interests, prior experiences, and preferences. For instance, participant P-07 expressed interest in “*squares and rectangles*” (Figure 6), which she related to shapes learned at school, while participant P-08 identified the lion figure as the most appealing element within the tropical-themed mural (Figure 7). Both participants repeatedly touched their preferred mural elements, which were positioned within their reach.

**Figure 8.**
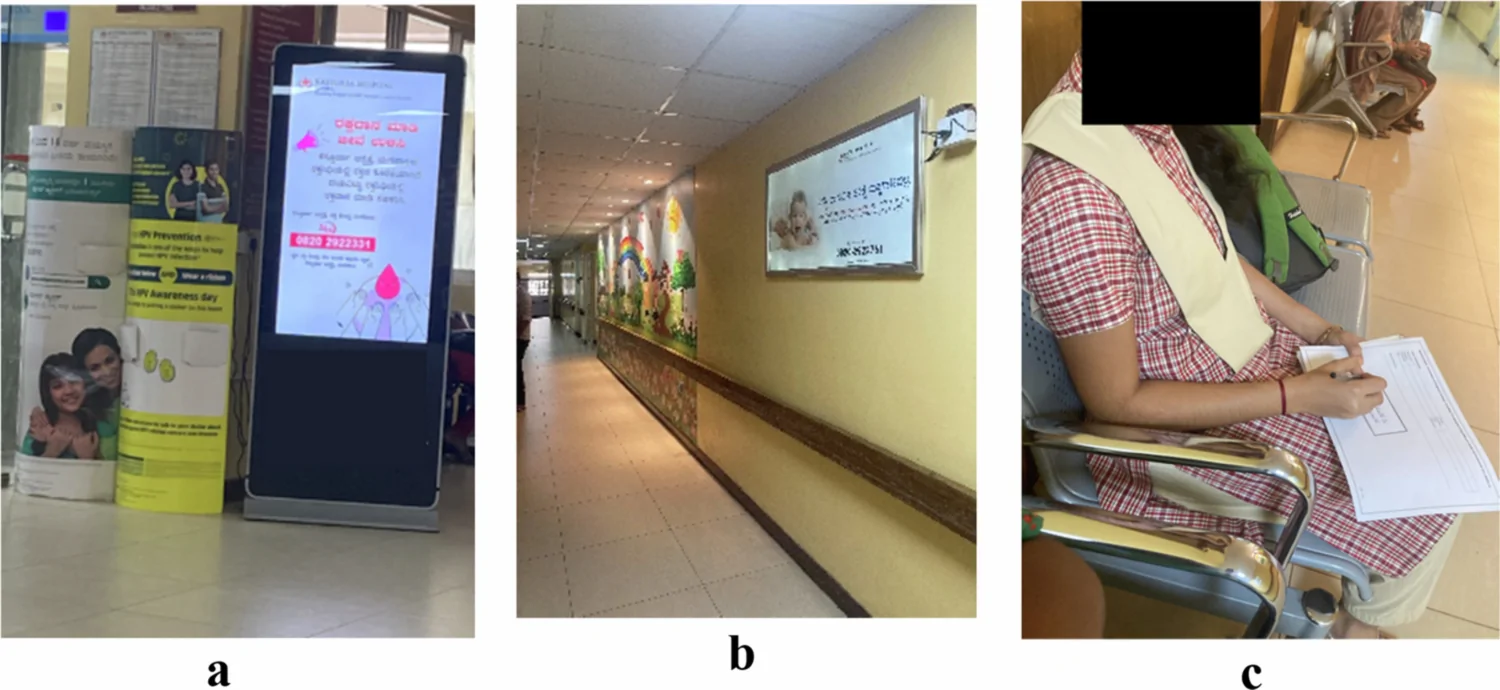
(a) Floor-mounted digital display standee near the entrance lobby. (b) Wall-mounted digital display panel opposite seating area. (c) Drawing of wall-mounted display panel by P-11 (modifications to drawing: none).

Additionally, digital display standee (Figure 8a) and wall-mounted display panel (Figure 8b) also functioned as familiar visual anchors within the lobby and corridor. Participants P-04 and P-09 collectively showed their interest in digital display standees and television screens and later represented them in drawings.

Participant P-04 described the floor-mounted digital display standee as a “*tall TV*”, illustrating how children interpreted unfamiliar hospital infrastructure through familiar media references. Similarly, participant P-11 identified the wall-mounted display board as a preferred feature, describing it as both visually engaging and informative. This preference was further illustrated in the participant's sketch (Figure 8c) and explained as follows:

✓ *“I like this display board…, colorful boards I like. Here, this is a white color display but at some other places there can be highlighted colorful boards which will make the place look better…. There can be a poster showing information required for sugar patients. Motivational and informative sentences for patients, slogan posters, educational posters, these would be good for sugar patients as they can see and understand.”* [P-11]

Familiar environmental engagement was evident across the majority of participants and was supported by convergent evidence from behavioural observations, drawings, and verbal responses. Although the specific environmental features varied across settings, recognizable play elements, mural features, and digital displays consistently emerged as preferred points of interaction.

Together, these findings indicated that familiar environmental features such as play elements, mural elements, and digital display panels were associated with familiar environmental engagement by encouraging recognition, tactile interaction, and sustained visual attention across the HPE. The consistency between children's observed behaviours, drawings, and verbal responses collectively indicated how recognizable spatial elements contributed to comfort and meaningful interaction within the everyday HPE. These patterns suggest that familiar environmental features may contribute to positive distraction by providing children with recognizable points of reference that support comfort and engagement within the HPE.

#### 5.3.2. Curiosity-driven environmental engagement

Curiosity-driven engagement refers to children' interaction with sensory-stimulating environmental features that capture attention and encourage exploration within the HPE. Curiosity-driven engagement was identified among a smaller group of participants, primarily within the lobby setting, where sensory-responsive environmental features attracted repeated attention and exploration. This theme had two sub-themes – engagement with sensory environmental features and exploration prompting peer interaction opportunities. Through this theme, auditory stimuli emerged as one such element that attracted children's interest and prompted interaction.

##### 5.3.2.1. Engagement with sensory environmental features.

**Figure 9.**
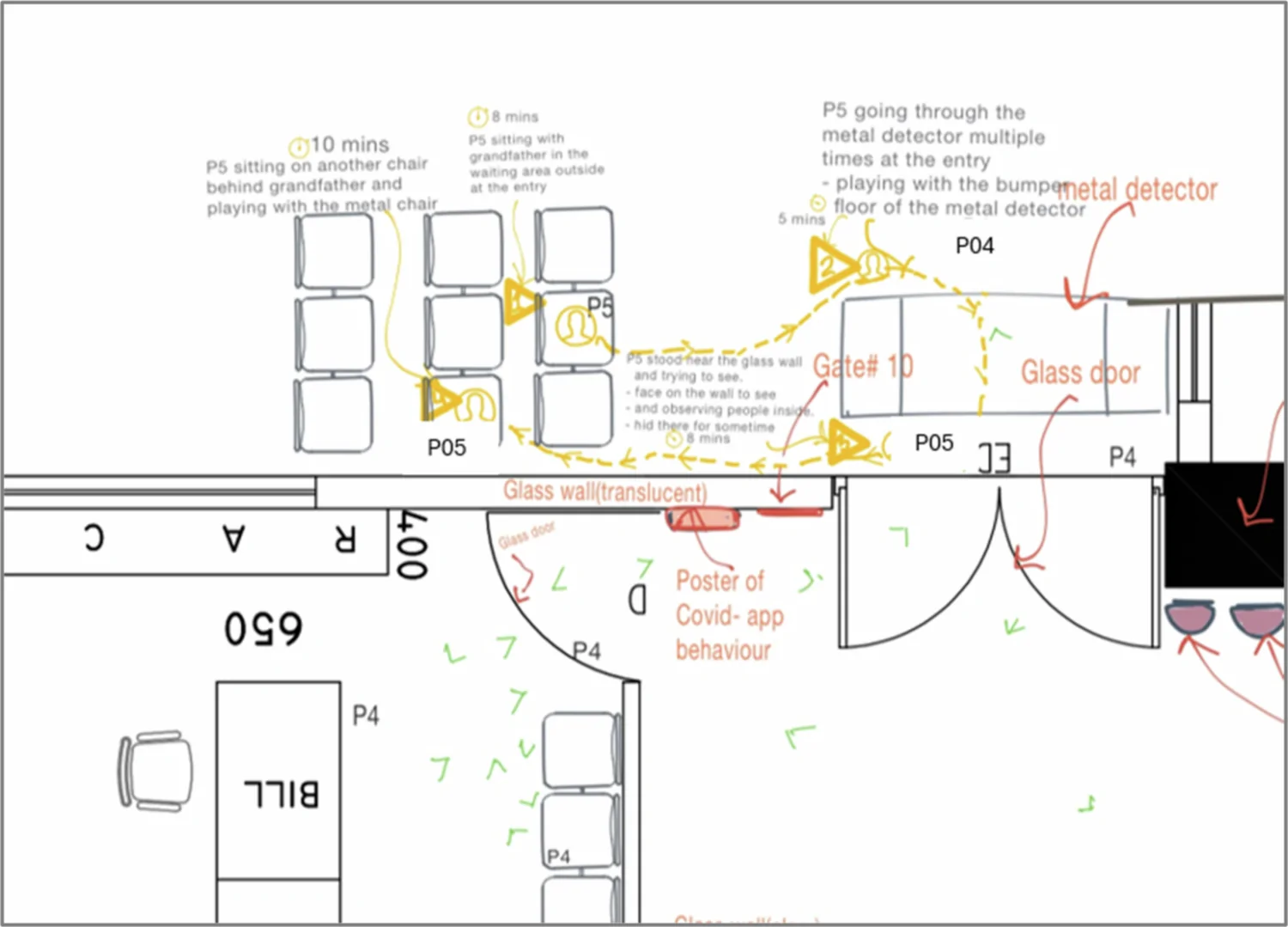
Behavioural observation map of participants P-04 and P-05 at the entrance lobby.

Auditory environmental stimuli emerged as an important feature for curiosity-driven engagements. Participants P-05, P-16 and P-17 showed sustained interest in the beeping sound produced by the metal detector located near the lobby of the hospital. As illustrated in the observation map (Figure 9), P-04 and P-05 repeatedly moved near the metal detector and spent approximately 70% of the observation period within this zone. This interest was further reflected in participants' drawings, where P-04, P-09, and P-17 represented the digital display standee and television (Table VIII), suggesting that these visually and auditorily engaging features remained salient beyond the immediate observation period.

Additionally, during verbal elicitation participants expressed a preference for media-related features. Participant P-04 described the digital display standee as a “*tall TV*,” whereas participants P-09 and P-17 identified the television as a preferred environmental feature, reinforcing the convergence of observations, drawings, and verbal responses (Table VIII).

##### 5.3.2.2. Exploration prompting peer interaction opportunities

A metal detector, although primarily a functional utility feature of the built environment, acted as a source of curiosity-driven environmental engagement for many participants (P-04, P-05, P-16, P-17). In addition, the elevated platform and responsive beeping sound further encouraged repeated movement behaviours. Crossing the platform activated the beeping sound reinforcing children's exploration interaction with this environmental feature.

Observation data of participant P-05 also indicated that such engagement occasionally supported spontaneous peer interaction (with participant, P-04) opportunities, indicating that the auditory stimulus not only captured attention but also encouraged social interaction within the setting.

Together, these findings indicate that responsive auditory features within the HPE may contribute to curiosity-driven environmental engagement by encouraging repeated movement, exploration, and occasional peer interaction. From the perspective of supportive design theory, such sensory-responsive features may operate as a positive distraction that redirects children's attention toward exploration and interaction within the healthcare setting.

#### 5.3.3. Inspirational environmental engagement

Inspirational environmental engagement refers to children's engagement with everyday elements that stimulate imagination, learning and future aspiration within the HPE. In this study, certain everyday functional elements prompted children to interpret the setting beyond its immediate context and relate them to their personal interests and future roles. This theme had two sub-themes – learning-oriented engagements and meaning making beyond clinical context.

##### 5.3.3.1. Learning-orientated engagement.

**Figure 10.**
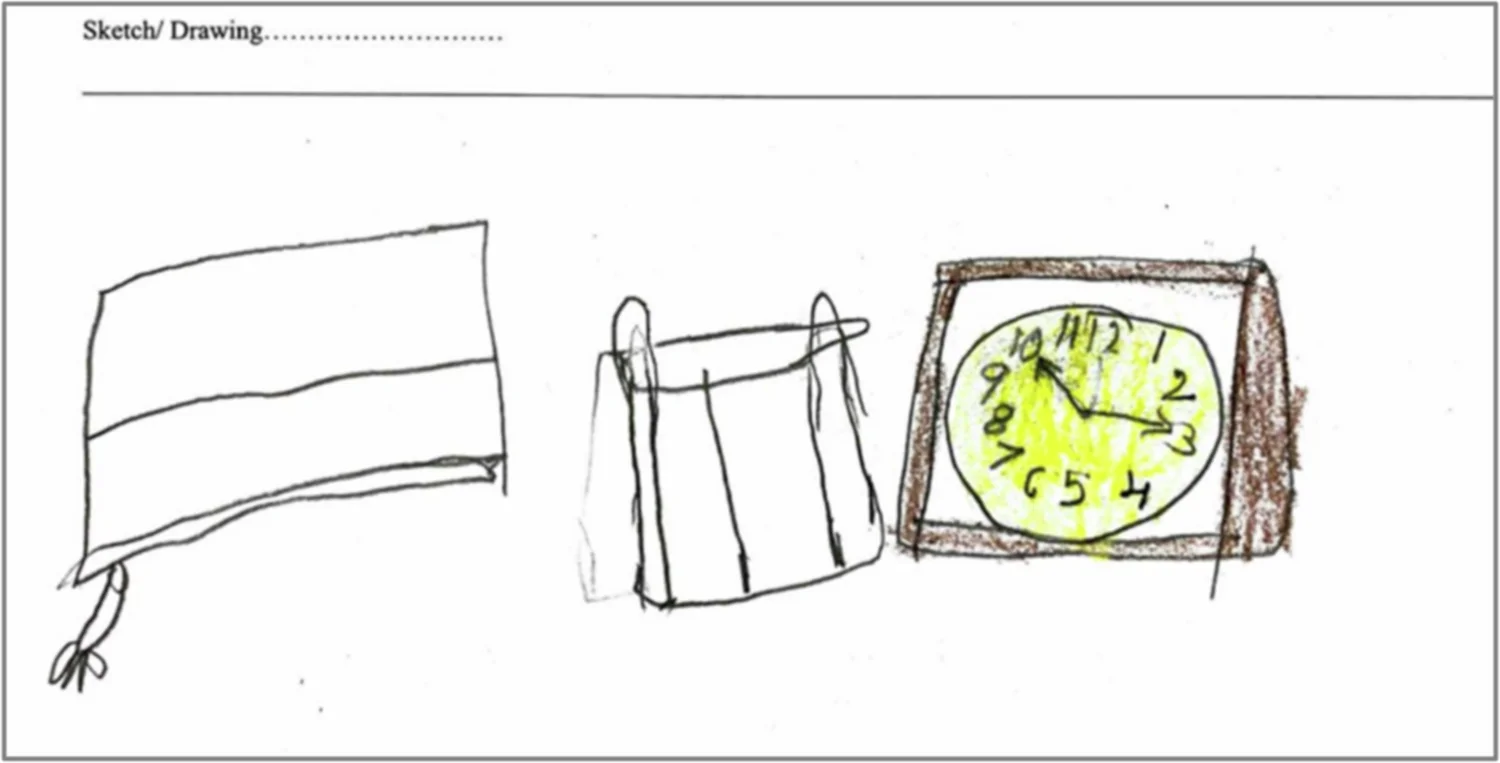
Drawing of a clock and other objects by P-12 (modifications to drawing: none).

Learning-orientated engagement with functional objects emerged as a form of inspirational environmental engagement, where children interpreted everyday elements as opportunities for exploration within the healthcare setting. For instance, behavioural observation showed that participant P-12 spent time observing the wall-mounted clock. This interaction was subsequently reflected in the participant's drawing (Figure 10) and explained during verbal elicitation: “*I have drawn that clock. Because there are numbers in it. That's why.”* The convergence of these data sources suggests that the clock became a point of learning-orientated engagement rather than merely a functional object, illustrating how everyday functional elements within the HPE may encourage incidental learning while shifting children's attention beyond the immediate clinical context.

##### 5.3.3.2. Meaning-making beyond clinical context.

**Figure 11.**
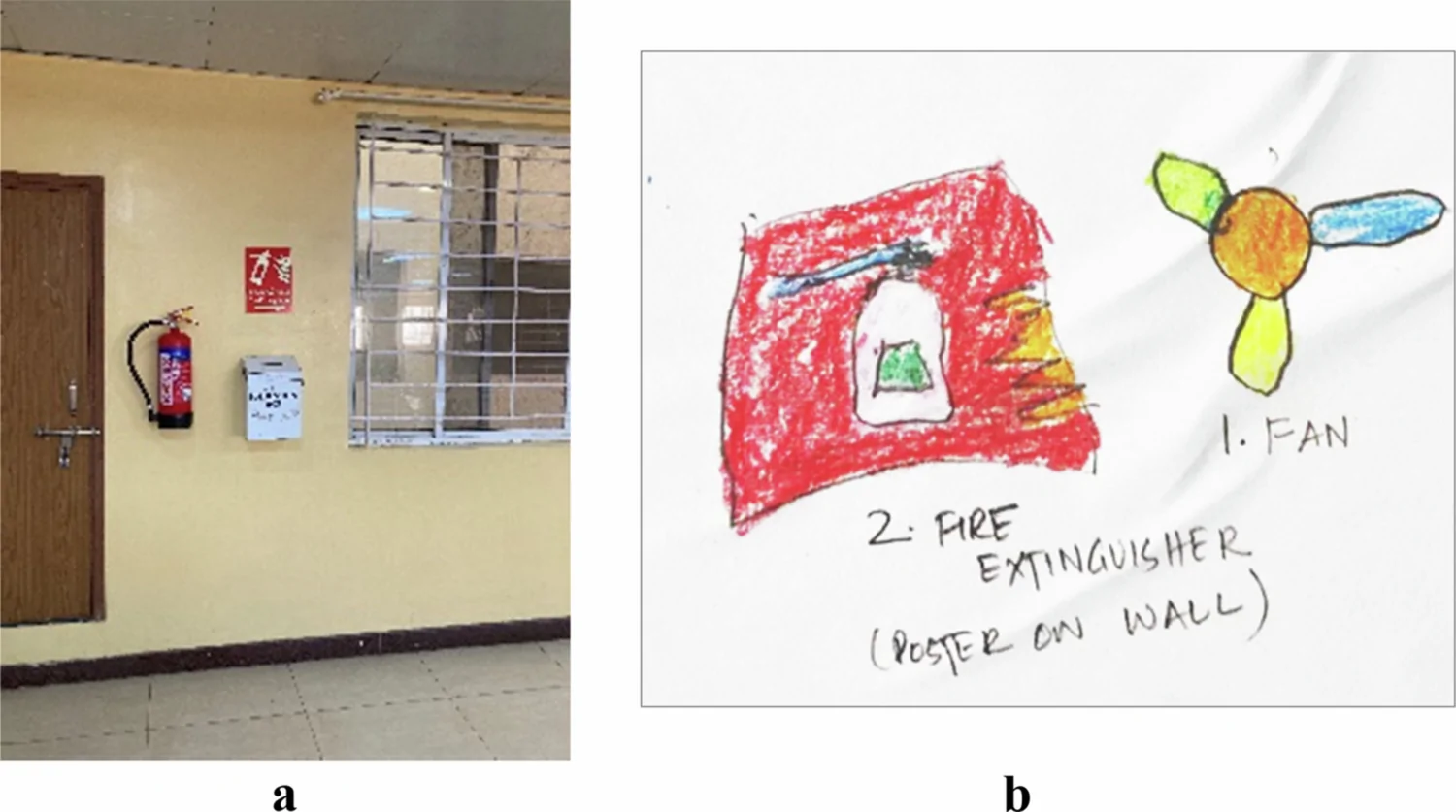
(a) Wall-mounted fire extinguisher in the waiting area. (b) Drawing of fire extinguisher and colourful fan by P-06 (modifications to drawing: none).

Visual environmental features were identified as sources of inspirational environmental engagement making meaning beyond the clinical context. Participant P-06 repeatedly observed the fire extinguisher in the waiting area (Figure 11a), subsequently represented it in the drawing activity (Figure 11b), and associated it with the aspiration to become a firefighter during verbal elicitation.

✓ *“I like red color… and I want to become a firefighter when I grow up. Because they are like heroes*” [P-06]
✓ *“I like the white coat, and I want to become a doctor, when I grow big.”* [P-22]

Likewise, participant P-22 associated the doctor's white coat with the aspiration to become a doctor, illustrating how children extended the meanings of everyday healthcare features beyond their immediate functional purpose.

Collectively, the observations, drawings, and verbal elicitations indicated that children interpreted environmental features in ways that extended beyond their immediate functional purpose, suggesting inspirational engagement through imagined future roles, meaning making, and learning-oriented interpretations.

### 5.4. Synthesis of multi-method qualitative approach

Findings from the three complementary data sources demonstrated how children's behavioural interactions with environmental features were reflected in their drawings and verbal responses across the four healthcare settings. This convergence of behavioural observations, drawings, and verbal elicitations provided a triangulated understanding of children's engagement with environmental features within everyday HPE. However, not all observed behaviours were reflected in children's drawings; some emerged through verbal elicitations, highlighting the complementary contribution of each method in capturing children's experiences within the HPE.

**Figure 12.**
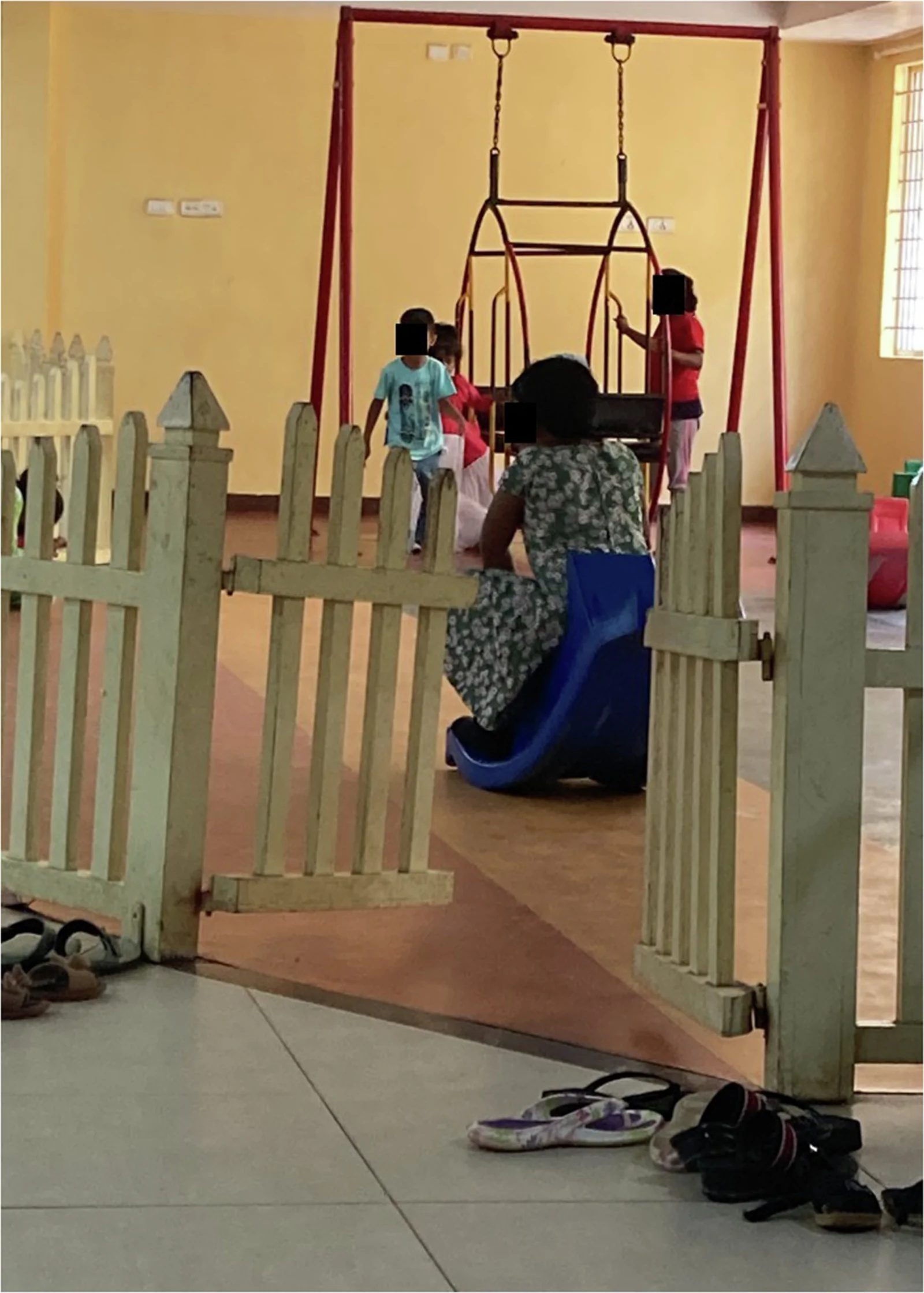
Wooden partition.

**Figure 13.**
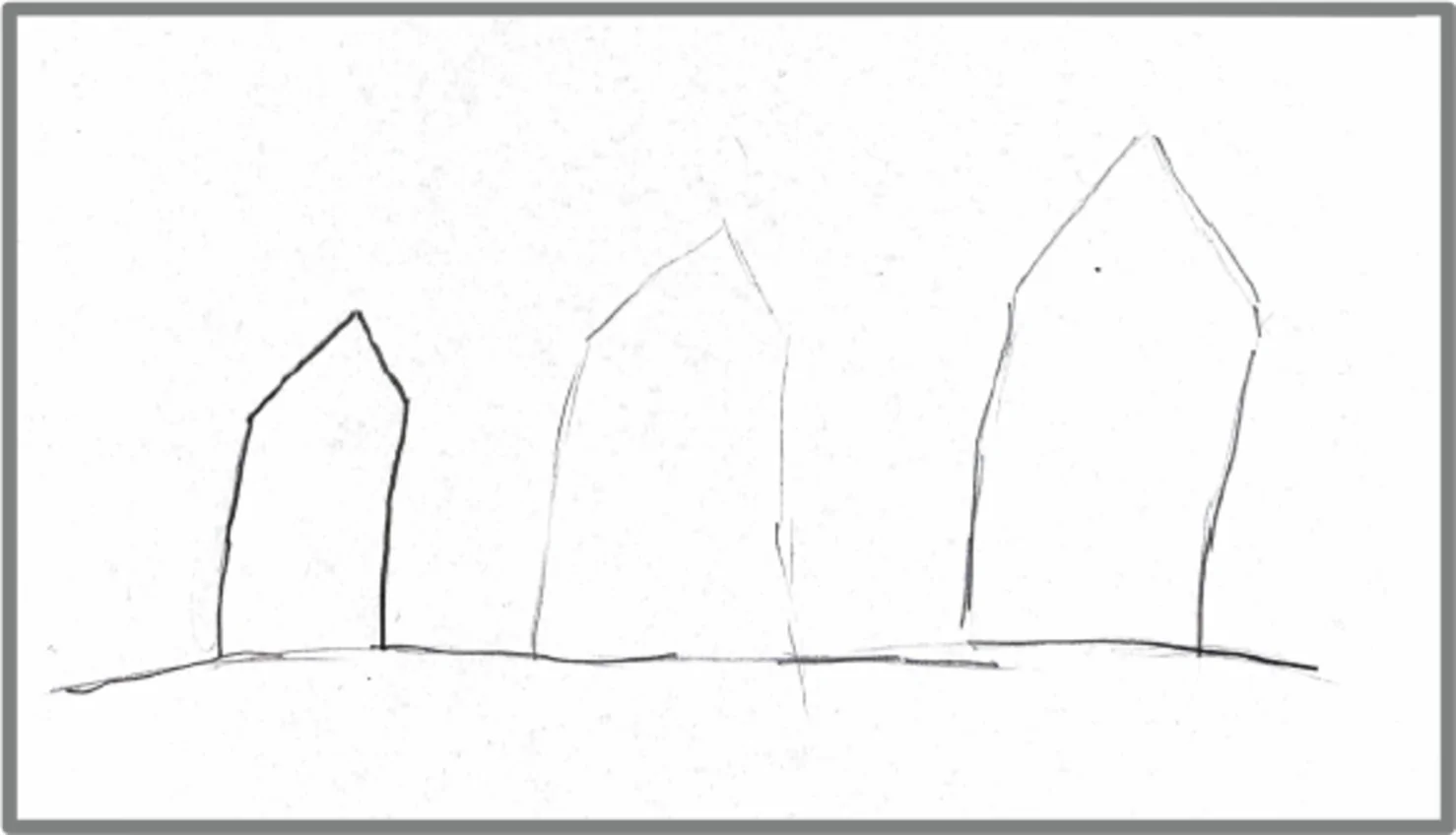
Drawing by P-01.

**Figure 14.**
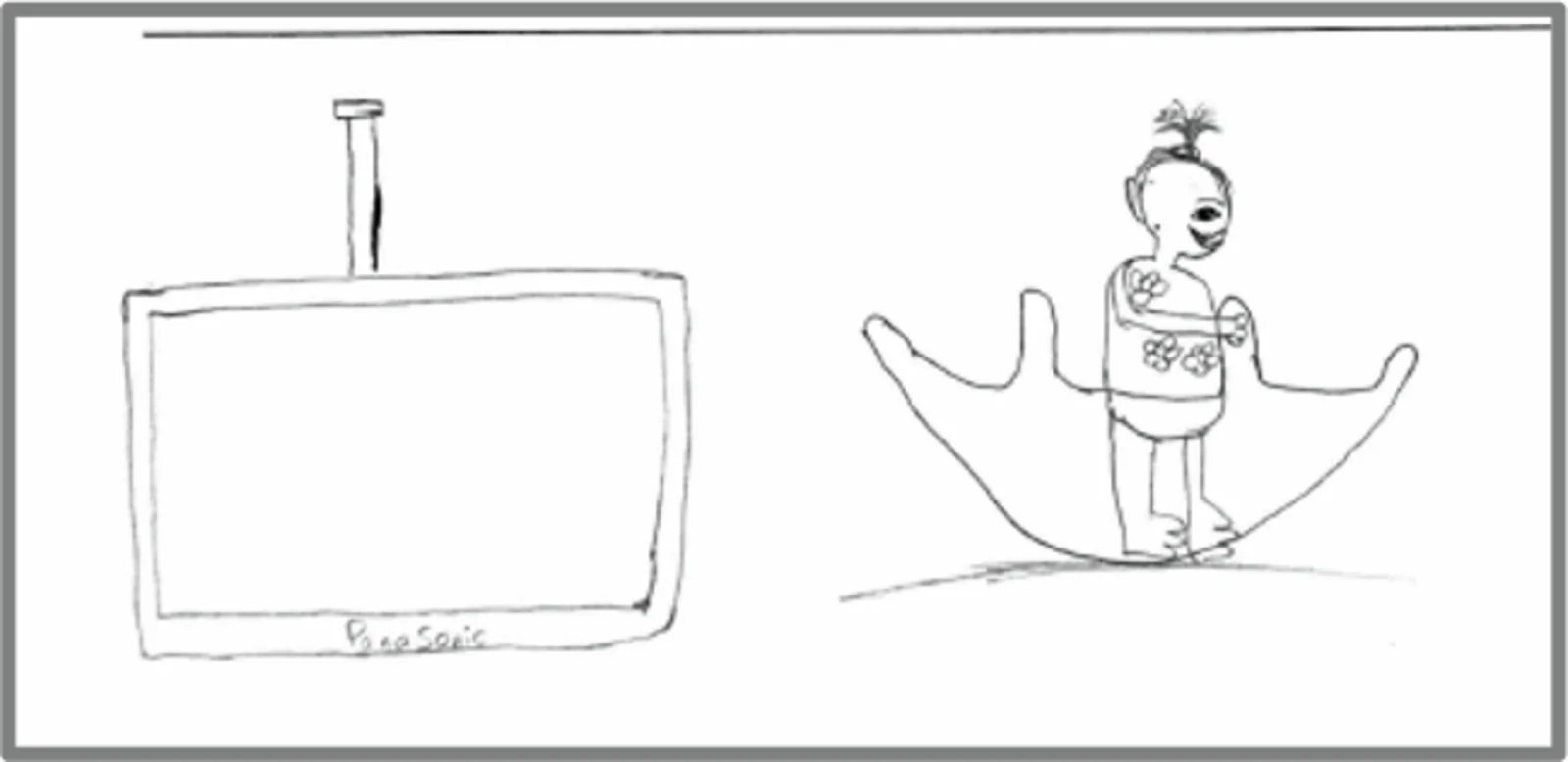
Drawing by P-13.

**Figure 15.**
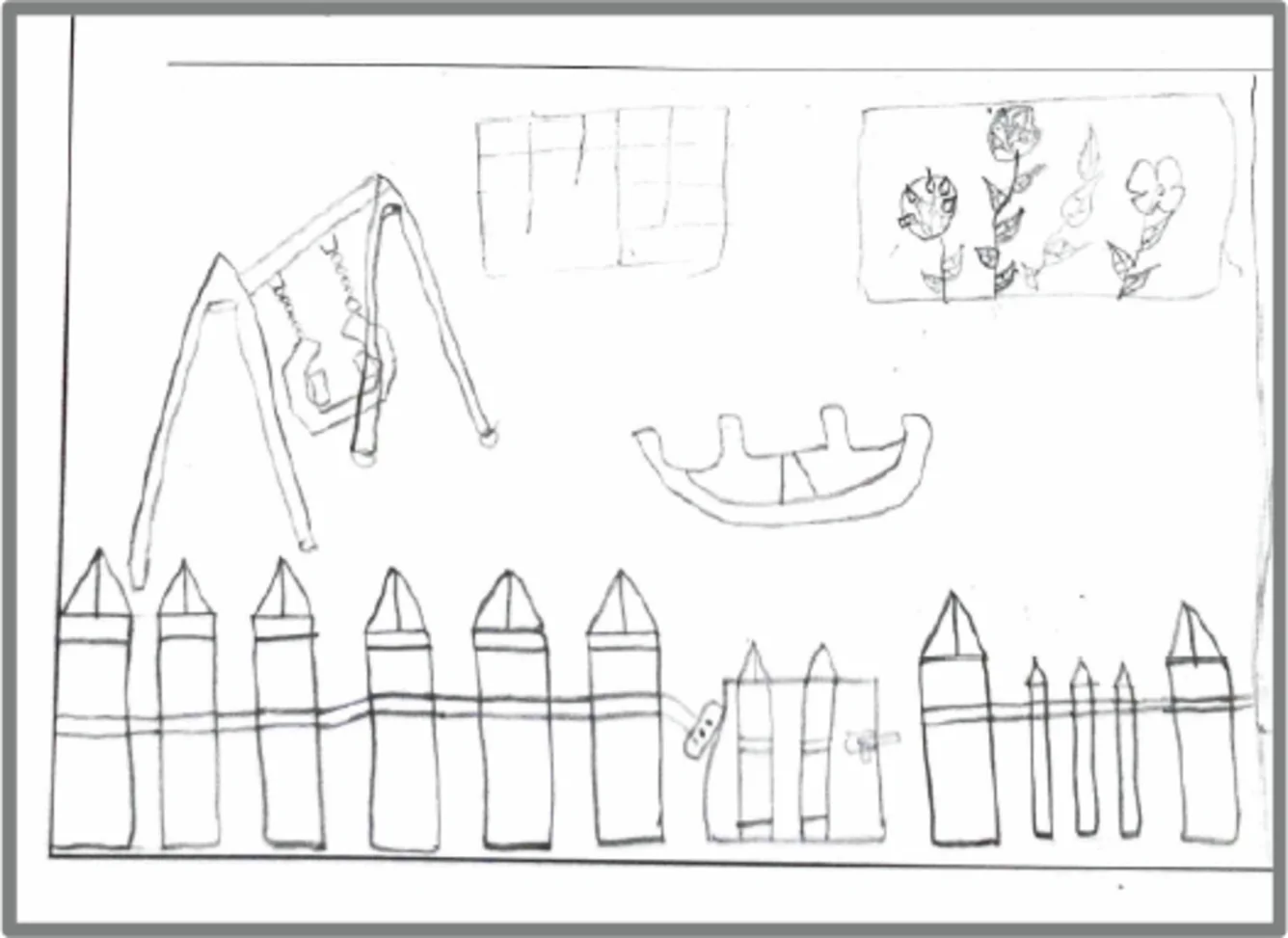
Drawing by P-24.

**Figure 16.**
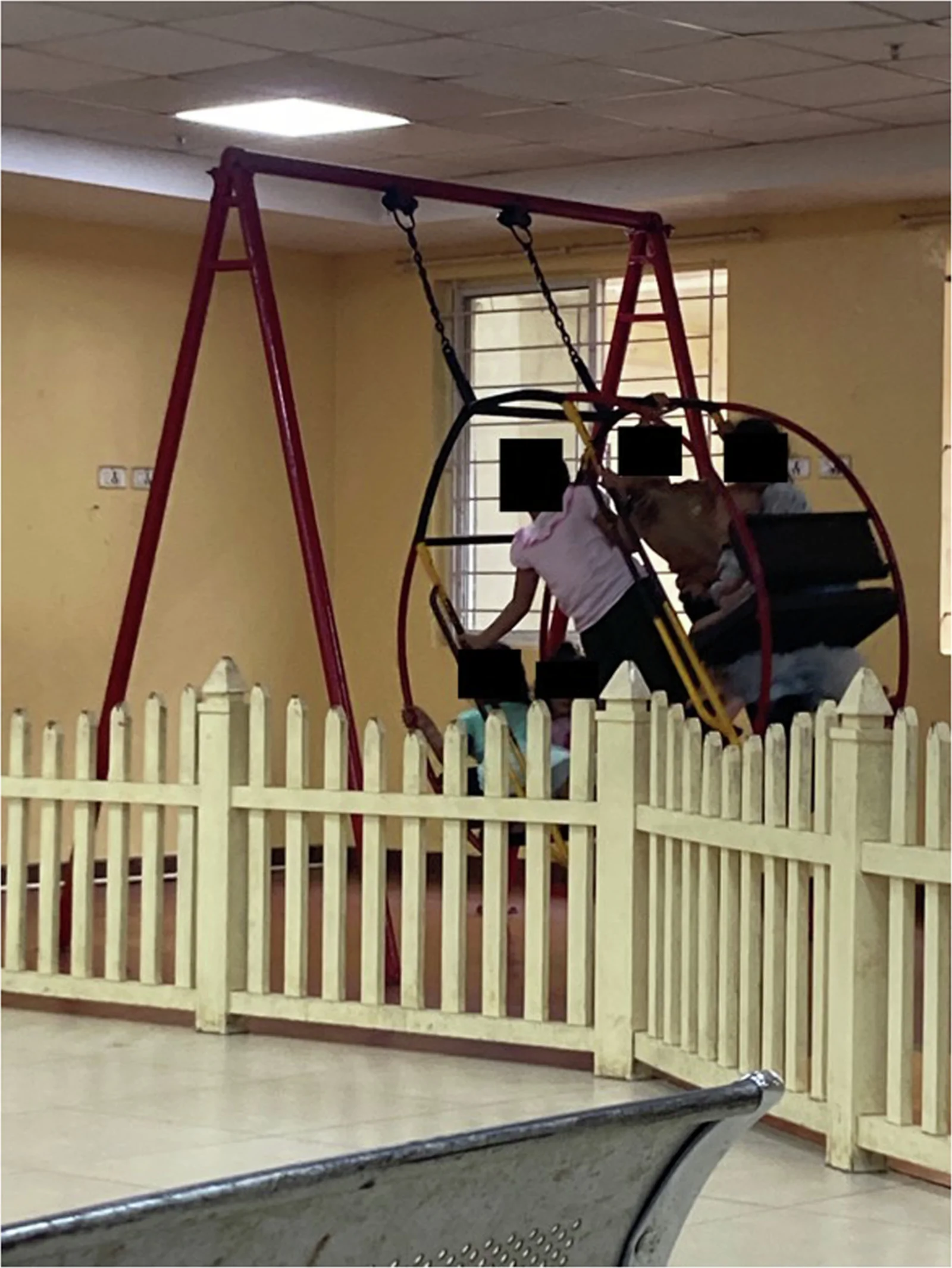
Swing.

**Figure 17.**
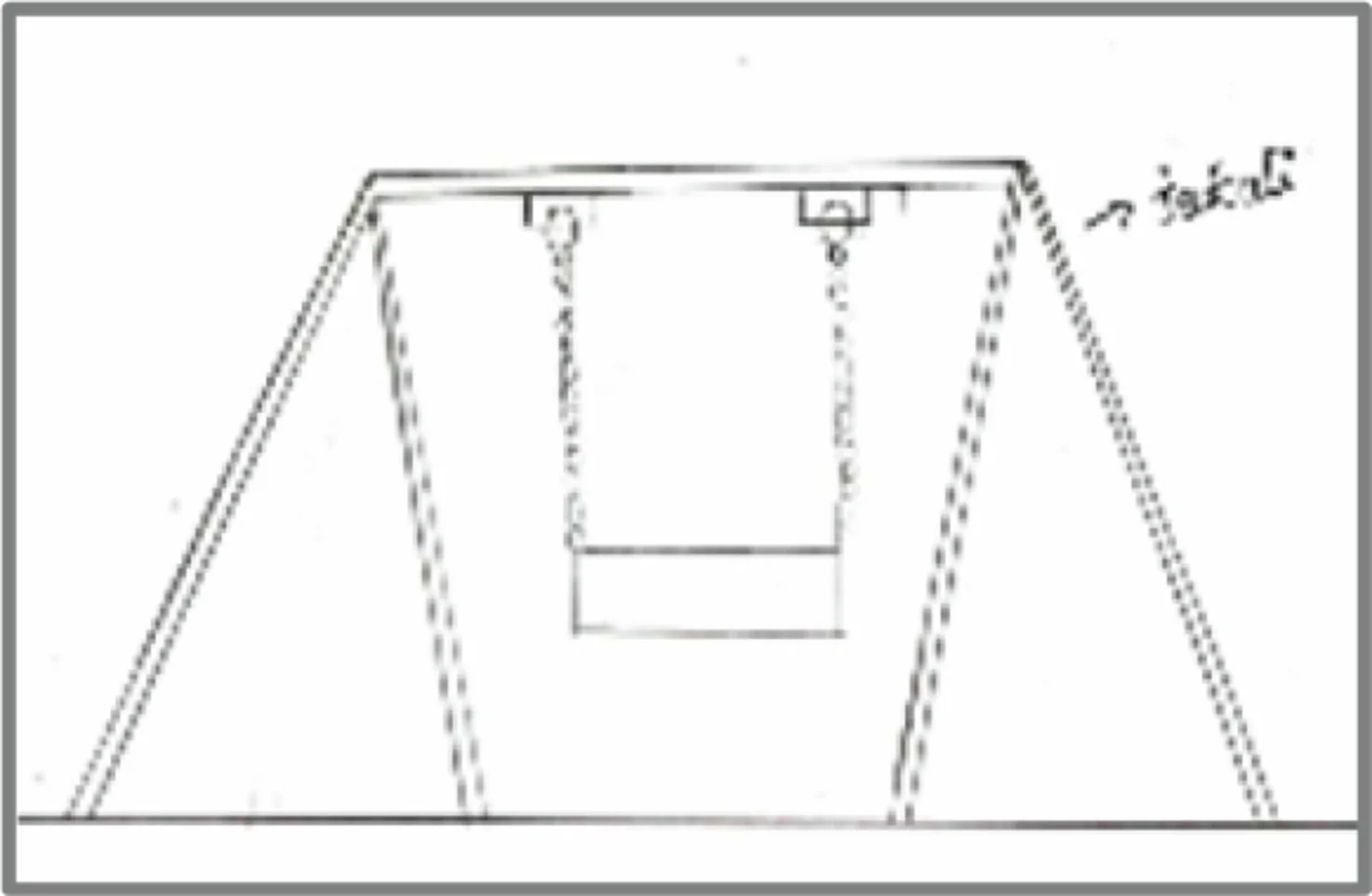
Drawing by P-03.

**Figure 18.**
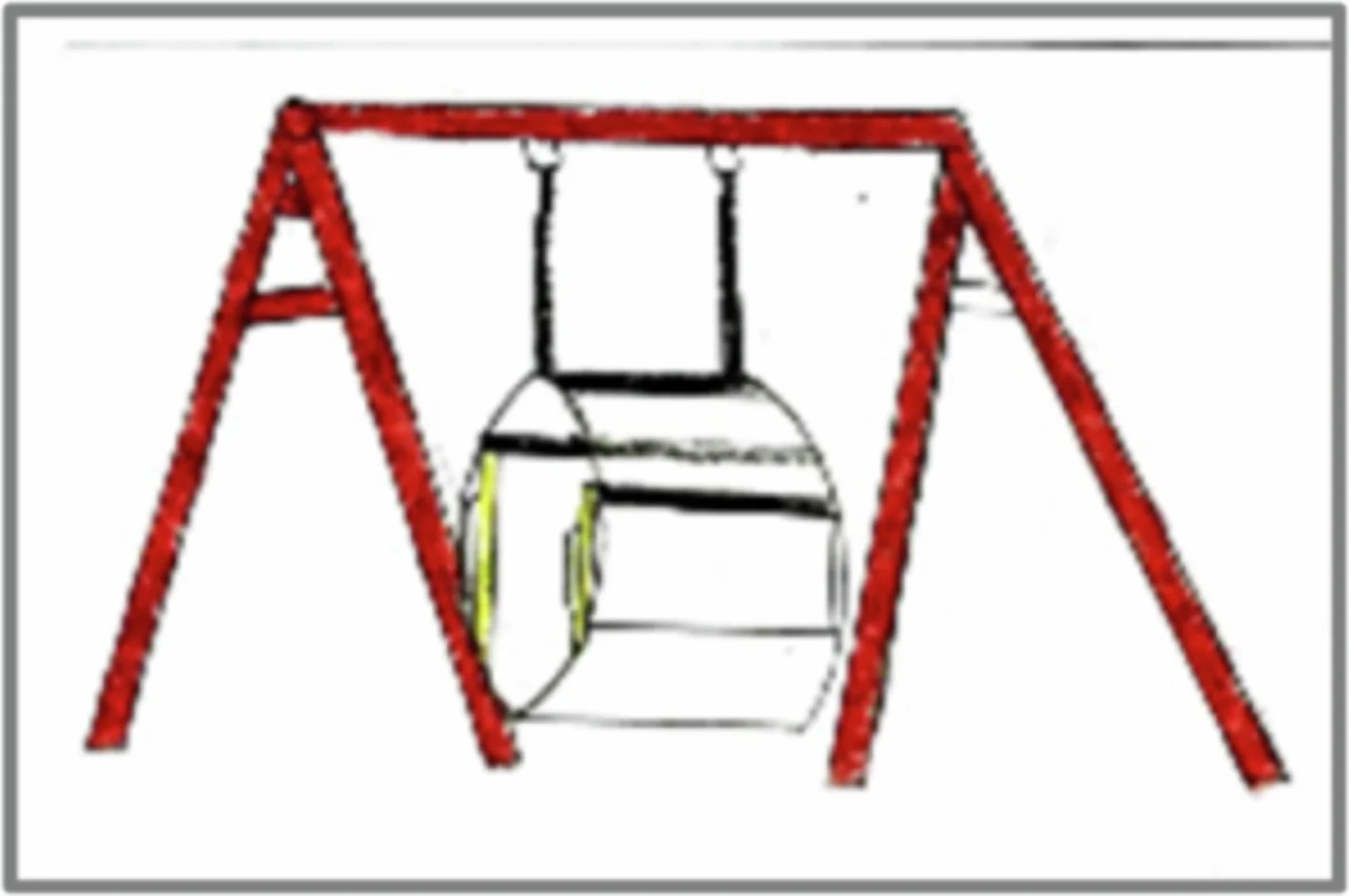
Drawing by P-15.

**Figure 19.**
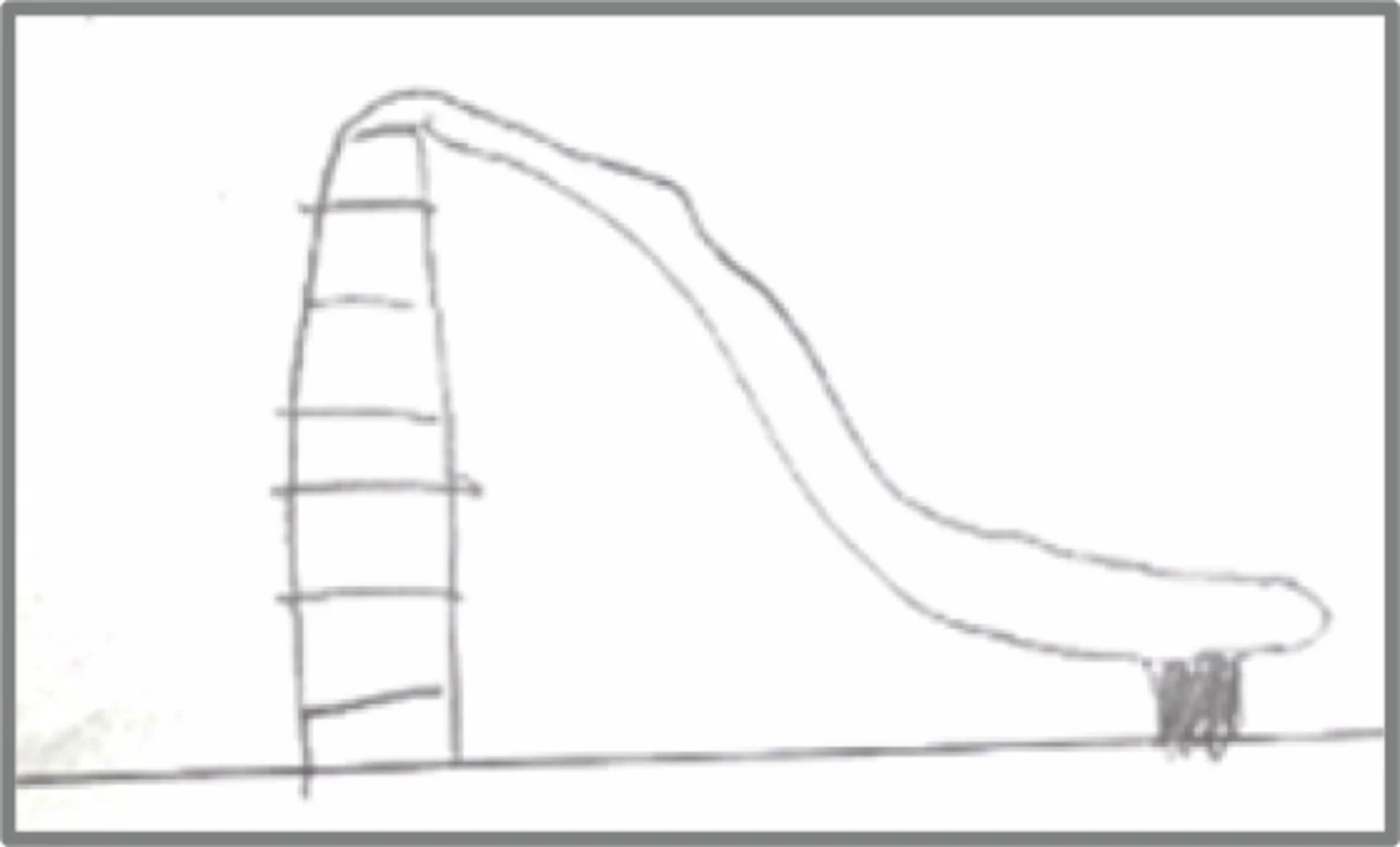
Drawing by P-23.

**Figure 20.**
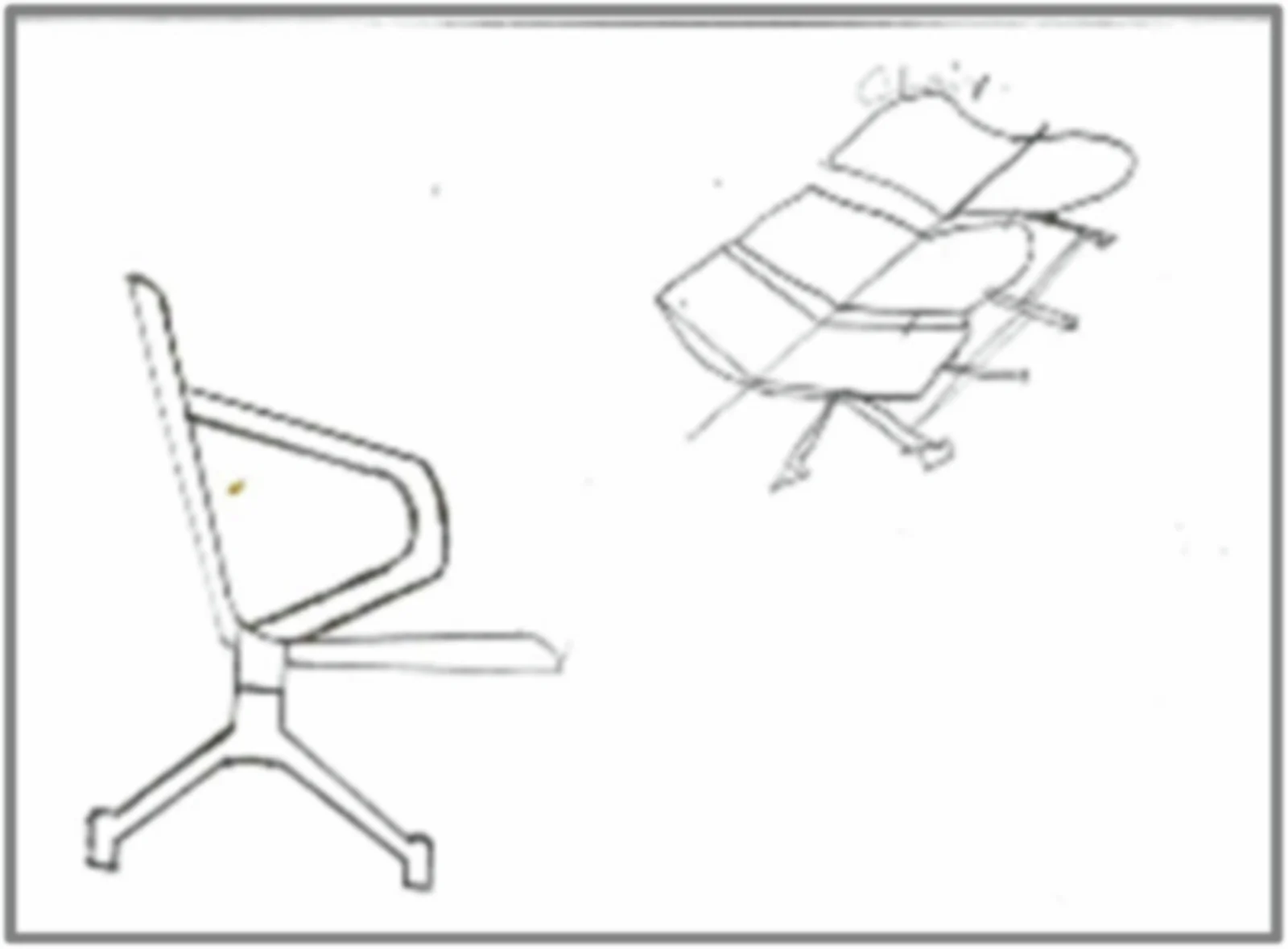
Drawing by P-18.

**Figure 21.**
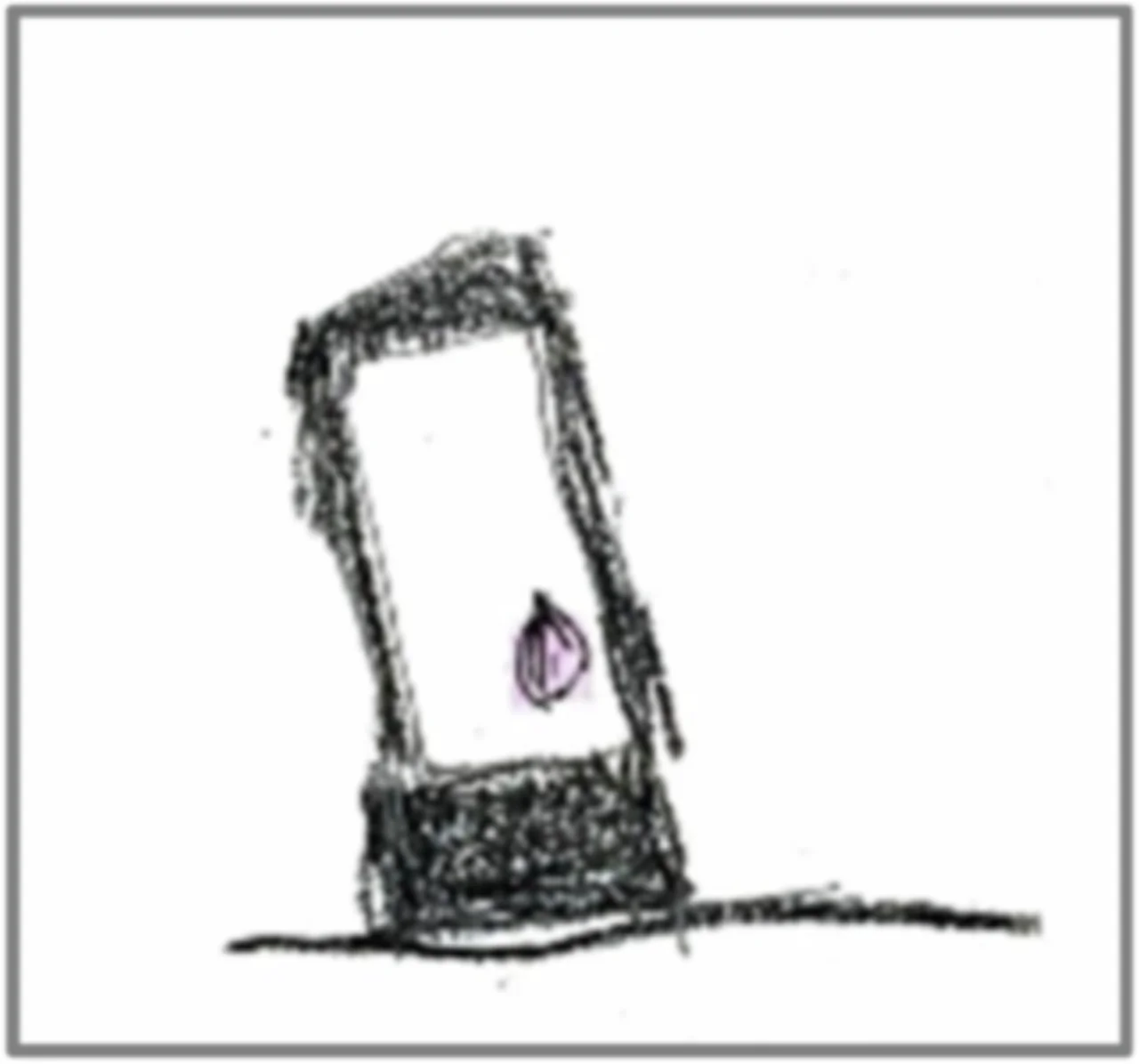
Drawing by P-04.

**Figure 22.**
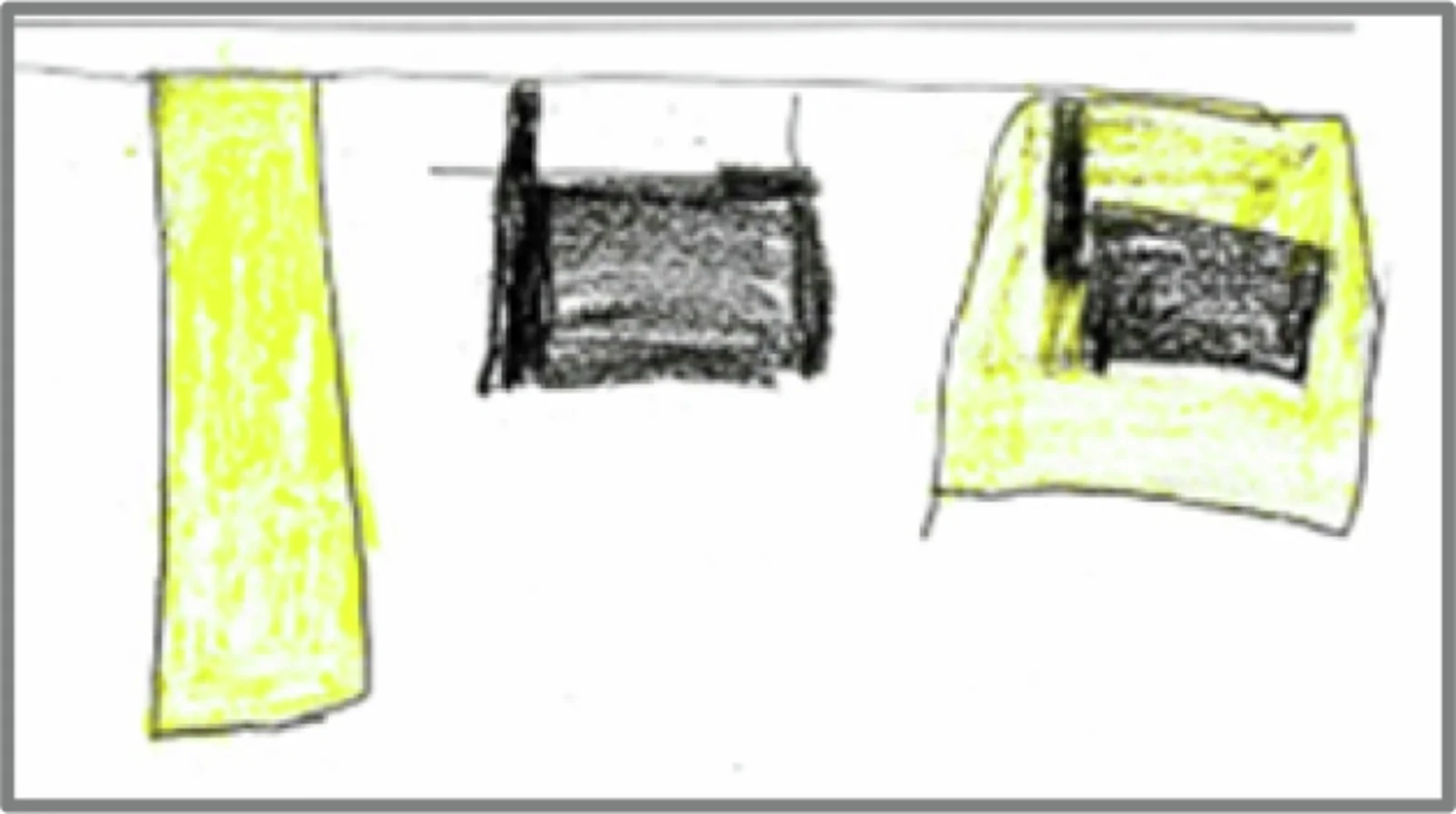
Drawing by P-09.

**Figure 23.**
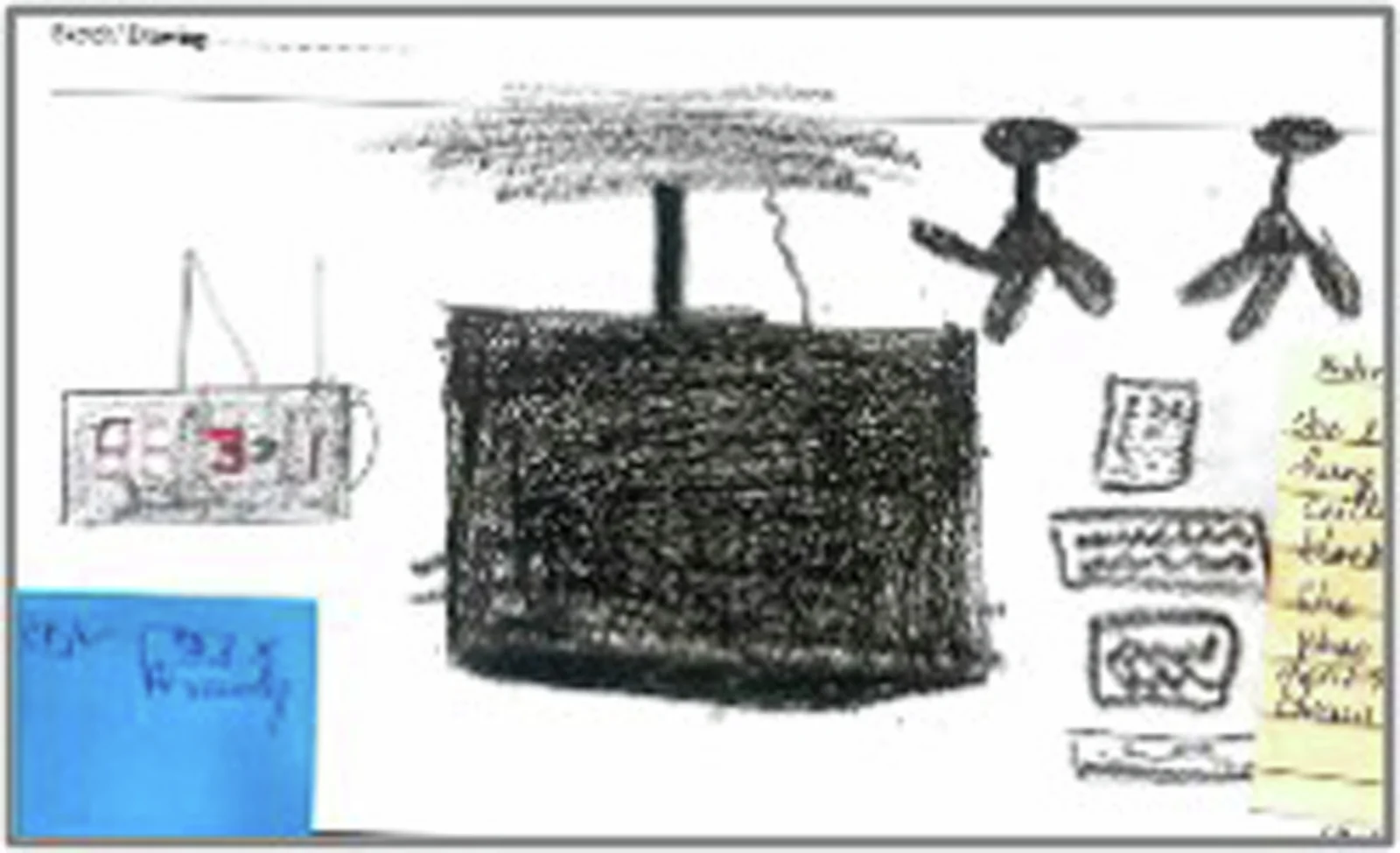
Drawing by P-17.

**Figure 24.**
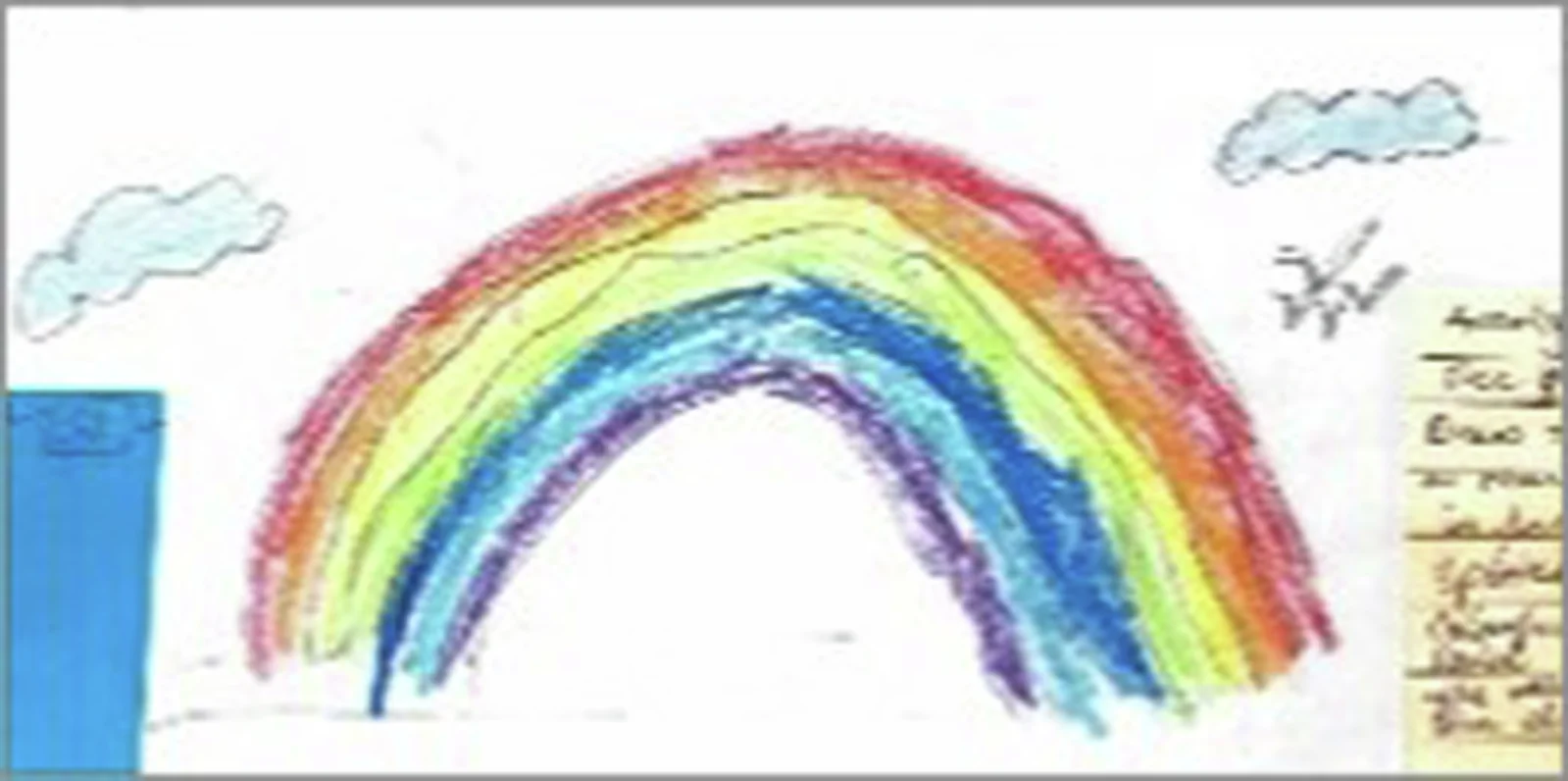
Drawing by P-10.

**Figure 25.**
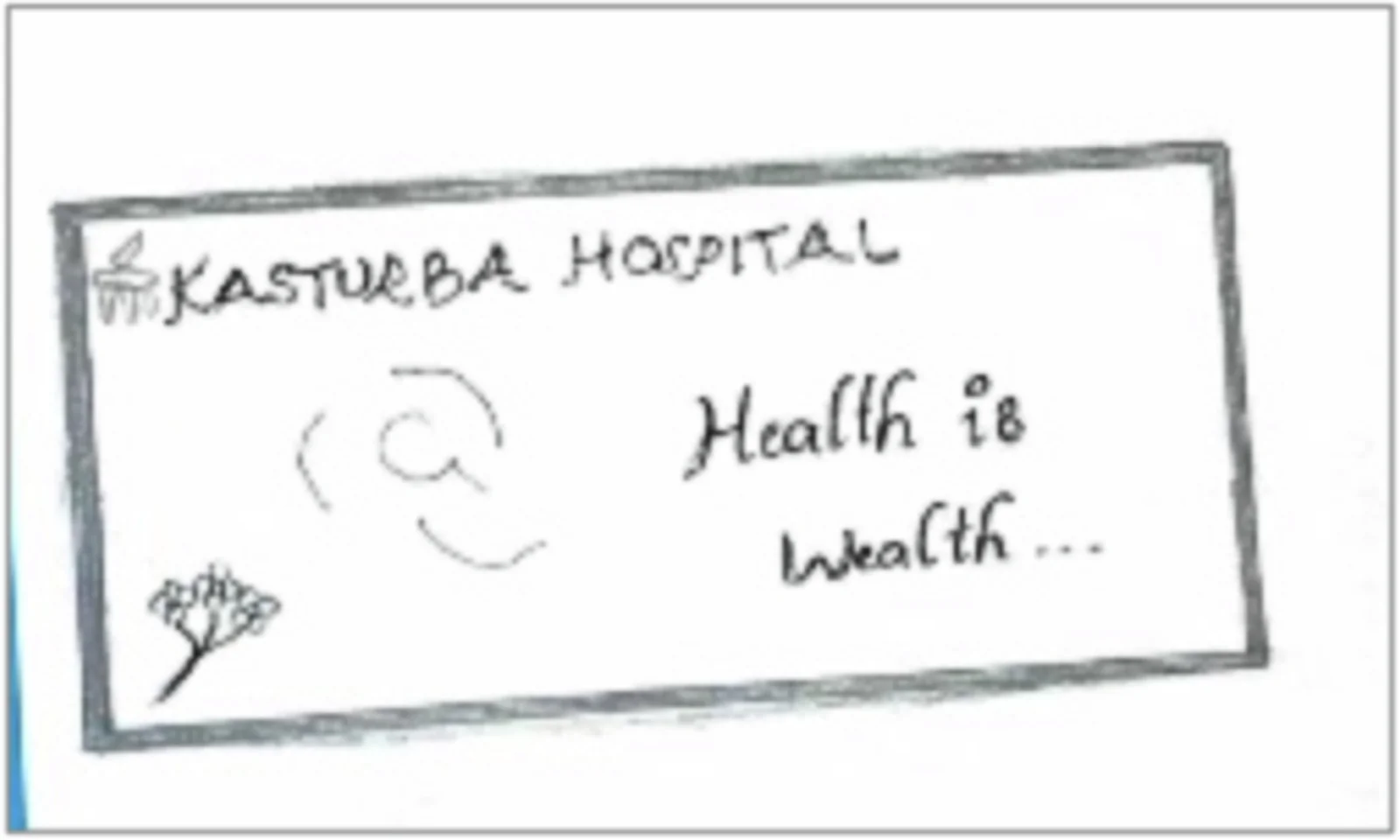
Drawing by P-11.

**Figure 26.**
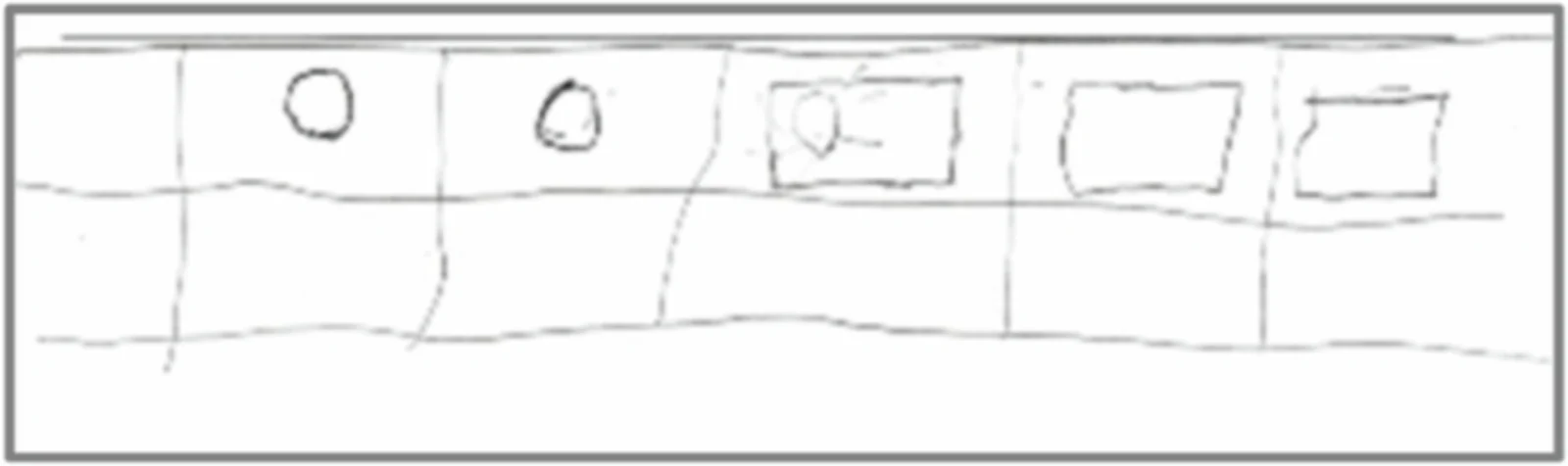
Drawing by P-20.

Table VIII summarizes illustrative examples of these relationships across the play area, waiting area, corridor, and lobby settings. In the play area, children interacted with elements such as swings, slides, and wooden railings, which were subsequently reflected in their drawings and described as preferred play features. In the waiting area, children explored mural walls and adjacent play elements that were later reproduced in drawings and identified as visually engaging features. Similarly, in the corridor and lobby, digital display panels and mural graphics attracted children's attention and were reflected in both drawings and verbal responses. Together, these patterns reflected an association between children's behavioural engagement with environmental features and their visual and verbal interpretations across the healthcare settings (Figures 12–26).

**Figure 27.**
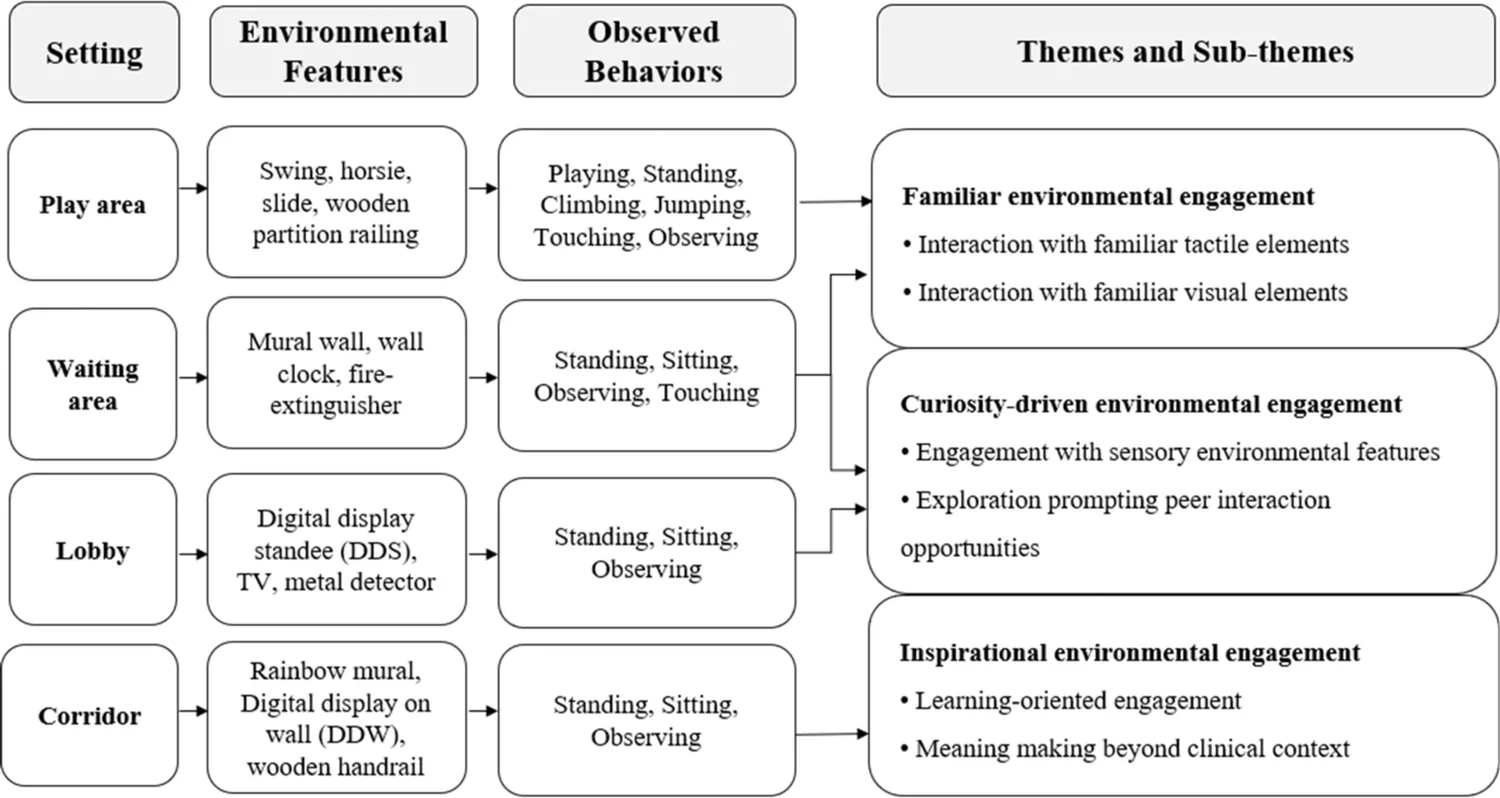
Synthesis of children's engagement across the four healthcare settings examined in the study (source: authors).

Table VIII provides a synthesis of the triangulated findings, linking children's behavioural engagement with environmental features and the corresponding forms of environmental engagement identified across the four healthcare environments. Building on this synthesis, Figure 27 visually integrates these relationships by illustrating how behavioural engagement patterns, environmental features, and children's experiential responses are interconnected across the four healthcare settings.

Figure 27 summarizes the relationships among children's behavioural engagement patterns, the environmental features with which they interacted, and the themes identified through the multi-method qualitative approach. Active behaviours in the play area were associated with interactive elements such as swings, toys, and wooden railings, supporting familiar environmental engagement. In the waiting area and lobby, observing and touching behaviours related to mural walls, clock, digital display boards, and the metal detector contributes to familiar, inspirational, and curiosity-driven forms of environmental engagement. The identified themes represented recurring patterns across participants, although not all children engaged with the same environmental features or expressed similar preferences. Differences were observed in the elements that attracted attention, reflecting individual interests, developmental characteristics, and situational factors. Overall, the integration of observations, drawings, and verbal elicitations enabled a richer understanding of children's experiences and engagement within everyday HPE.

## 6. Discussion

### 6.1. Children's engagement within everyday healthcare settings

The study sought to understand how children engaged with everyday HPE through a multi-method qualitative approach combining behavioural observations, drawings and verbal elicitations. By considering children's actions alongside their visual and verbal expressions, the study provides a richer interpretation of how spatial characteristics and sensory dimensions shape children's experiences within the HPE. This resonates with previous research that suggests children actively interpret and respond to environmental conditions through interaction, highlighting the importance of studying their behaviours within naturally occurring spatial contexts (Ferguson et al., 2013; Spencer & Blades, 2006). Furthermore, multi-method qualitative approaches are increasingly recommended in child-centered research because they capture both observable interaction patterns and children's subjective interpretations of space (Clark, 2008).

Children's engagement across the observed settings was associated with environmental affordances for movement, interaction, and sensory exploration. Spaces containing play equipment and interactive elements were commonly associated with more active behaviours, such as playing, jumping, and climbing, whereas settings with fewer features were commonly associated with passive behaviours such as sitting, standing, and observing. However, these behaviours should not necessarily be interpreted as indicators of limited engagement. Within healthcare settings, sitting, standing, and observing may also reflect social and situational factors that are beyond the scope of the present study, such as waiting periods, fatigue, caregiver instruction, health status or previous hospital experiences (Coyne, 2006; Zhang & Wyness, 2024). Recent pediatric healthcare research similarly suggests that children's experiences are shaped by a combination of environmental, social, and contextual factors (Goel et al., 2024; Sathyanarayanan & Caldas, 2025; Yu et al., 2024). Overall, while environmental affordances appeared to influence opportunities for interaction, children's passive behaviours should be understood within a broader context of the healthcare environment. These observations are consistent with research suggesting that physical environmental characteristics may influence how children interact with and experience their surroundings (Evans, 2021; Ferguson et al., 2013).

Although mural graphics and digital display elements contributed to visual interest in transitional spaces such as corridors, they were associated with comparatively limited active engagement. This observation provides a nuanced perspective on previous evidence from healthcare design literature suggesting that visual enhancements, artwork, and aesthetically appealing interiors may contribute positively to children's hospital experiences (Ulrich et al., 2008; Watts & Wilson, 2009). Recent studies have highlighted the value of visual, artistic, and child-friendly design features in supporting pediatric well-being and environmental satisfaction (Goel et al., 2024; Mohd Zairul and Noor Hafizah, 2025). More specifically, the observations from the present study suggest that visual features alone may not be sufficient to encourage sustained interactions; however, deeper engagement appears to occur when environmental features also provide opportunities for tactile exploration, movement, or interaction. The findings further suggest that environmental features positioned at children's eye level and within accessible reach facilitate visual attention, tactile exploration, and active engagement.

Conversely, play areas and waiting zones, where visual interest was combined with opportunities for movement, tactile interaction, and exploration, were associated with a wide range of active behaviours. This observation is consistent with recent evidence suggesting that play opportunities, interactive environmental features, and child-centered design may encourage children's engagement and positive healthcare experiences (Babbu & Haque, 2023; Bahrami et al., 2025; Yu et al., 2024). Furthermore, this interpretation is aligned with contemporary pediatric healthcare design perspectives that emphasize multi-sensory, flexible, and patient-centered environments that may support diverse developmental and experiential needs (Sathyanarayanan & Caldas, 2025; Yu et al., 2024).

Overall, the insights from the present study indicate that visual stimulation may be more meaningful when combined with opportunities for movement, tactile interaction, and exploration. This observation is consistent with child-centered design perspectives emphasizing multi-sensory engagement rather than single-modality visual interventions within healthcare environments (Melles et al., 2020).

### 6.2. Children's experiences within healthcare settings

The thematic outcomes further suggest that children engaged with environmental features through familiar, curiosity-driven, and inspirational forms of engagement. Familiar environmental engagement is associated with interaction with recognizable play elements, mural graphics, and digital display media that support orientation and comfort within unfamiliar hospital settings. Similarly, Lambert et al. (2014) reported that recognizable spatial features may help children interpret healthcare environments and contribute positively to their environmental experience. Studies have also shown that children often interpret unfamiliar environmental features through familiar everyday references and understand new experiences by relating them to existing cognitive schemas (Coyne, 2006; Piaget, 1950; Spencer & Blades, 2006). These observations are also consistent with recent child-centered healthcare design research highlighting the importance of familiar, playful, and visually relatable environmental features in supporting children's comfort and engagement during healthcare visits (Babbu & Haque, 2023; Bahrami et al., 2025).

Curiosity-driven environmental engagement is associated with responsive sensory elements within the HPE. These sensory elements encourage repeated movement and occasional peer interaction, illustrating how even functional infrastructure elements can act as novel sensory stimuli within the HPE. Although such behaviours may reflect curiosity-driven active engagement, they should also be considered within the broader context of healthcare safety and functionality (Bock et al., 2021; Ulrich et al., 2008). Research suggests that responsive sound-based cues may naturally attract attention and encourage exploratory engagement, particularly among children in an unfamiliar environment (Bock et al., 2021; Goel et al., 2024; Razzaghipour et al., 2024; Sathyanarayanan & Caldas, 2025; Ulrich et al., 2008). Such exploratory behaviours may also reflect children's natural tendency to explore sensory stimuli within their environments, a process widely recognized in developmental research (Cosco et al., 2010; Coyne, 2006; Piaget, 1950; Razzaghipour et al., 2024).

Inspirational environmental engagement is reflected in children's learning-oriented and future-oriented thinking beyond everyday functional objects such as clocks, signboards, fire extinguisher and doctor's white coat. These associations extend beyond the immediate clinical context, suggesting that built environments may communicate meaning symbolically as well as functionally (Rapoport, 1990; Spencer & Blades, 2006). More specifically, some children constructed meanings from such environmental features and connected them with imagined future roles (Vygotsky, 2004). These observations resonate with studies suggesting that children actively construct meanings, personal narratives through developmental learning processes during hospital experiences (Clark, 2008; Spencer & Blades, 2006; Vygotsky, 2004).

The multi-method qualitative approach provides a more comprehensive understanding of children's experiences in HPE. The integration of behavioural observations, drawings, and verbal elicitations strengthens the interpretive depth of the study through methodological triangulation. Likewise, previous research highlights that combining visual and verbal child-centered methods improves understanding of children's experiences by capturing both explicit and implicit forms of expression (Clark, 2008; Greig et al., 2013). Drawings provide access to children's perspectives that may not be easily articulated verbally, while accompanying elicitation helps reduce ambiguity in interpretation and supports analytical reliability (Drake & Winner, 2013). These findings reinforce recommendations that multi-method qualitative approaches are essential for capturing children's perspectives within healthcare research contexts (Angelöw & Psouni, 2025; Barrett & Twycross, 2018; Hunleth et al., 2022).

More broadly, the insights are consistent with healthcare design research suggesting that multi-sensory environments may contribute positively to children's experiences during hospital visits (Bock et al., 2021; Razzaghipour et al., 2024; Ulrich et al., 2008). However, the findings should additionally be interpreted within the cultural context of Indian healthcare settings, where family members often play an active role in accompanying and supporting children during healthcare visits (Coyne, 2006; Zhang & Wyness, 2024). This suggests that children's experiences are shaped not only by environmental design but also by social and relational influences.

Taken together, the three forms of engagement represent different yet interconnected ways through which children interacted with everyday healthcare environments. Viewed collectively through the lens of supportive design theory, these forms of engagement suggest that healthcare environments may support children's experiences through multiple pathways, ranging from familiarity and exploration to meaning making and aspiration. More importantly, this study illustrates how integrating observation and behavioural mapping with drawings and verbal elicitation provides a structured approach for examining these forms of engagement within healthcare environments. By foregrounding children's behavioural, visual, and verbal perspectives, the study contributes to a deeper understanding of how everyday environmental features may support familiar, curiosity-driven, and inspirational forms of engagement within healthcare settings.

The three identified forms of engagement-familiar, curiosity-driven, and inspirational engagement-are broadly consistent with previous pediatric healthcare research emphasizing familiarity, exploration, and active engagement (Bock et al., 2021; Lambert et al., 2014; Watts & Wilson, 2009). However, rather than examining these environmental attributes as isolated design features, the present study suggests that children engage with healthcare environments through interconnected processes of familiarity, exploration, and meaning making. Collectively, these findings provide a more nuanced understanding of children's environmental engagement within everyday healthcare settings.

## 7. Conclusions

This study examined how children engaged with everyday healthcare environments through a multi-method qualitative approach integrating observations, drawings, and verbal elicitations. The insights derived from the study suggest that children actively perceive and respond to environmental features across healthcare settings through their behaviours, engagement, and emotional responses. Furthermore, integrating behavioural observation with art-based drawings and verbal elicitations provides a robust and nuanced approach for capturing both the explicit and implicit dimensions of children's experiences within the HPE.

Healthcare facilities in India serve large and diverse patient populations, often requiring healthcare environments to balance clinical functionality and resource constraints with child-friendly design considerations (Goel et al., 2024; National Health Authority, 2022; Patel et al., 2015). Within this context, environmental features that support familiarity, sensory engagement, and positive distraction may play an important role in shaping children's experiences during healthcare visits. Importantly, children's engagement with healthcare environments is also likely influenced by broader social and cultural factors, including family involvement, healthcare expectations, and previous experiences with hospital settings.

From a theoretical standpoint, this study deepens the field of environment–behaviour research by extending the application of supportive design theory to children's everyday healthcare experiences. Supportive design theory conceptualizes positive distraction as an environmental resource that helps reduce stress (2001; Ulrich et al., 2008; Ulrich, 1991). The findings from the present study suggest that its effectiveness depends not only on the presence of environmental elements but also on children's opportunities for active engagement and multisensory interaction with those features. This indicates that positive distraction is not solely a characteristic of the physical environment; rather, it emerges through the dynamic interaction between children and their surroundings.

The study identifies three interconnected forms of engagement—familiar, curiosity-driven, and inspirational—that explain how children interpret and derive meaning from healthcare environments. These forms of engagement position children as active meaning makers of the physical environment whose spatial experiences extend beyond most adult perspectives. The study further contributes by suggesting that visual features alone may not be sufficient to sustain meaningful engagement; rather, meaningful engagement is strengthened when opportunities for tactile exploration, movement, and interaction are present. This highlights the need for multi-sensory environmental engagement within child-responsive healthcare. Importantly, the study highlights that the accessibility of environmental features, particularly those positioned within children's physical reach, plays a critical role in enabling sensory exploration and meaningful engagement. Collectively, these findings refine supportive design theory by suggesting that, in pediatric healthcare environments, positive distraction is shaped not only by environmental attributes but also by children's active engagement, multisensory interaction, and the accessibility of design elements.

While this study offers meaningful insights into children's experiences within hospital settings, several limitations should also be acknowledged. To begin, although children's drawings served as a rich medium for understanding children's perspectives, the interpretation remains inherently subject to the researcher's interpretation. To minimize this limitation, drawings were interpreted alongside participants' verbal responses to support contextual interpretation. In addition, the relatively small sample size within each healthcare setting (ranging from five to eight participants per setting), together with the single institutional context, may limit the broader transferability of the findings. Finally, although participants ranged from 5 to 18 years of age, the study was not designed to undertake age-specific analyses but rather to explore children's environmental engagement across a broad developmental spectrum.

Future research could address the limitations through larger sample sizes and studies conducted across multiple healthcare contexts, enabling comparisons across developmental age groups, healthcare contexts, and cultural settings. Such studies could also extend to specialized healthcare environments such as emergency departments, intensive care units, and diagnostic settings, where spatial conditions and emotional demands differ considerably from everyday hospital areas. Further research could explore the experiences of specific pediatric populations, including children with chronic illnesses, disabilities, and neurodivergent conditions, whose experiences and interactions with the healthcare environment may differ from those explored in this study. Methodologically, future studies could use longitudinal approaches to interpret how children's experiences evolve over time and across healthcare encounters. Such studies may provide a deeper understanding of how healthcare environments can better respond to children's experiential needs and inform design strategies that support their emotional health, well-being, and participation in healthcare settings.

Ultimately, the study responds to the enduring observation that *"the environments of children are not always environments for children…."* by offering empirical evidence to support the design of healthcare settings that genuinely reflect children's experiences, needs, and everyday interactions. In doing so, the study shifts the focus from environments that are merely occupied by children to environments that are meaningfully shaped around children, their needs, and their ways of experiencing and engaging with healthcare spaces.

## Supplementary Material

Supplementary material Supplementary Material 4 SI 4.docx

Supplementary material Supplementary Material 2 SI 2.docx

Supplementary material Supplementary Material 3 SI 3.docx

Supplementary material Supplementary Material 1 SI 1.docx

## Data Availability

Due to ethical and confidentiality considerations associated with research involving children, the raw data generated during the study is not publicly available. However, de-identified supplementary materials, including the overview of physical settings, sample observation map, verbal elicitation guide, and behavioural mapping template, are provided as Supplementary information for reference.

## Abbreviation

HPE: Healthcare physical environment

## References

[cit0001] Agarwal, M. , Sehgal, V. , & Ogra, A. (2021). Creating a child-friendly environment: An interpretation of children’s drawings from planned neighborhood parks of Lucknow City. *Societies* , *11* (3), 80. 10.3390/soc11030080

[cit0002] Aldridge, M. , & Wood, J. (1997). Talking about feelings:Young children's ability to express emotions. *Child Abuse & Neglect* , 21(12), 1221–1233. 10.1016/S0145-2134(97)00097-5 9429773

[cit0003] Angelöw, A. , & Psouni, E. (2025). Participatory research with children: From child-rights based principles to practical guidelines for meaningful and ethical participation. *International Journal of Qualitative Methods* , *24* , 10.1177/16094069251315391

[cit0004] Babbu, A. H. , & Haque, M. (2023). A framework for the design of pediatric healthcare environment using the Delphi technique. *Ain Shams Engineering Journal* , *14* (5). 101975. 10.1016/j.asej.2022.101975

[cit0005] Bahrami, B. , Kariminejad, S. , & Erfani, G. (2025). Child-friendly design in paediatric healthcare environments: Exploring children’s preferences for nature, space, and play. *Journal of Asian Architecture and Building Engineering* , *25* , 3102–3115. 10.1080/13467581.2025.2524532

[cit0006] Barrett, D. , & Twycross, A. (2018). Data collection in qualitative research. *Evidence-Based Nursing* , *21* , 63–64. 10.1136/eb-2018-102939 29858278

[cit0007] Bock, E. P. , Nilsson, S. , Jansson, P.-A. , Wijk, H. , Alexiou, E. , Lindahl, G. , & Degl’Innocenti, A. (2021). Literature review: Evidence-based health outcomes and perceptions. *Journal of Pediatric Nursing* , *61* , e42–e50. 10.1016/j.pedn.2021.04.013 33875322

[cit0008] Braun, V. , & Clarke, V. (2006). Using thematic analysis in psychology. *Qualitative Research in Psychology* , *3* , 77–101. 10.1191/1478088706qp063oa

[cit0009] Brooks, M. (2009). Drawing, visualisation and young children’s exploration of Big ideas. *International Journal of Science Education* , *31* (3), 319–341. 10.1080/09500690802595771

[cit0010] Clark, A. (2008). How to listen to very young children: The mosaic approach. *Child Care in Practice* , *7* (4), 333–341. 10.1080/13575270108415344

[cit0011] Cosco, N. G. , Moore, R. C. , & Islam, M. Z. (2010). Behavior mapping: A method for linking preschool physical activity and outdoor design. *Medicine and science in sports and exercise* , *42* , 513–519. 10.1249/MSS.0b013e3181cea27a 20068497

[cit0012] Coyne, I. (2006). Consultation with children in hospital: Children, parents’ and nurses’ perspectives. *Journal of Clinical Nursing* , *15* , 61–71. 10.1111/j.1365-2702.2005.01247.x 16390525

[cit0013] Creswell, J. W. (2014). *Research Design: Qualitative, Quantitative, and Mixed Methods Approaches* . CA: Thousand Oaks, CA: Sage Publications.

[cit0014] Denzin, N. K. (1978). *The Research Act: A Theoretical Introduction to Sociological Methods* . McGraw-Hill.

[cit0015] Drake, J. E. , & Winner, E. (2013). How children use drawing to regulate their emotions. *Cognition and Emotion* , *27* (3), 512–520. 10.1080/02699931.2012.720567 22963448

[cit0016] Einarsdóttir, J. (2007). Research with children: Methodological and ethical challenges. *European Early Childhood Education Research Journal* , *15* , 197–211. 10.1080/13502930701321477

[cit0017] Evans, G. W. (2021). The physical context of child development. *Current Directions in Psychological Science* , *30* (1), 41–48. 10.1177/0963721420980719

[cit0018] Ferguson, K. T. , Cassells, R. C. , MacAllister, J. W. , & Evans, G. W. (2013). The physical environment and child development: An international review. *International Journal of Psychology: Journal international de Psychologie* , *48* (4), 437–468. 10.1080/00207594.2013.804190 23808797 PMC4489931

[cit0019] Goel, S. , Mihandoust, S. , Joseph, A. , Markowitz, J. , Gonzales, A. , & Browning, M. (2024). Design of pediatric outpatient procedure environments: A pilot study to understand the perceptions of patients and their parents. *HERD: Health Environments Research & Design Journal* , *17* (2), 183–199. 10.1177/19375867231220398 38166516

[cit0020] Greig, A. , Taylor, J. , & MacKay, T. (2013). *Doing Research with Children: A Practical Guide* . SAGE Publications, Ltd. 10.4135/9781526402219

[cit0021] Guest, G. , Namey, E. E. , & Mitchell, M. L. (2013). Participant observation, *Collecting Qualitative Data: A Field Manual for Applied Research* (pp. 75–112). SAGE Publications, Ltd. 10.4135/9781506374680.n3

[cit0022] Hunleth, J. M. , Spray, J. S. , Meehan, C. , Lang, C. W. , & Njelesani, J. (2022). What is the state of children’s participation in qualitative research on health interventions? A scoping study. *BMC pediatrics* , *22* (1), 328. 10.1186/s12887-022-03391-2 35659206 PMC9166159

[cit0023] Kaelin, K. , & Okland, K. (2018). Buildings, barriers, and breakthroughs: Bridging gaps in the health care enterprise. *Nursing Administration* , *42* , 15–25. 10.1097/NAQ.0000000000000269 29194329

[cit0024] Lambert, V. , Coad, J. , Hicks, P. , & Glacken, M. (2014). Young children’s perspectives of ideal physical design features for hospital-built environments. *Journal of Child Health Care* , *18* (1), 57–71. 10.1177/1367493512473852 23423998

[cit0025] Malterud, K. , Siersma, V. D. , & Guassora, A. D. (2016). Sample size in qualitative interview studies: Guided by information power: Guided by information power. *Qualitative Health Research* , *26* (13), 1753–1760. 10.1177/1049732315617444 26613970

[cit0026] Melles, M. , Albayrak, A. , & Goossens, R. (2020). Innovating health care: Key characteristics of human-centered design. *International Journal for Quality in Health Care* , *33* , 37–44. 10.1093/intqhc/mzaa127 PMC7802070 33068104

[cit0027] Mohd Zairul, M. Z. , & Noor Hafizah, N. H. (2025). Current trends in art and design for paediatric wellness in built environments (2020–2025): A thematic review. *Frontiers in Pediatrics* , *13* , 2296–2360. 10.3389/fped.2025.1666934 PMC12832306 41602877

[cit0028] National Health Authority. ( 2022). Indian Public Health Standards (IPHS) Guidelines.

[cit0029] Nuvolari-Duodo, I. , Brambilla, A. , Ricciardi, G. E. , Dolcini, M. , & Capolongo, S. (2024). New requirements for post-COVID-19 hospital inpatient wards: Evidence, design recommendations and assessment tools. *Annali di Igiene* , *36* (2), 182–193. 10.7416/ai.2024.2601 38275084

[cit0030] O’Brien, B. C. , Harris, I. B. , Beckman, T. J. , Reed, D. A. , & Cook, D. A. (2014). Standards for reporting qualitative research: A synthesis of recommendations. *Academic Medicine* , *89* (9), 1245–1251. 10.1097/ACM.0000000000000388 24979285

[cit0031] O’Cathain, A. , Murphy, E. , & Nicholl, J. (2010). Three techniques for integrating data in mixed methods studies. *The BMJ* , *341* , 341–c4587. 10.1136/bmj.c4587 20851841

[cit0032] Patel, V. , Mazumdar-Shaw, K. , Kang, G. , Das, P. , Khanna, T. , & Divi, N. (2015). Health of the nation: India's healthcare system. *The Lancet* , *386* (10008), 2423–2424. 10.1016/S0140-6736(15)01288-7

[cit0033] Piaget, J. (1950). *The Psychology of Intelligence* . Routledge. 10.4324/9780203164730

[cit0034] Rapoport, A. (1990). *The Meaning of the Built Environment: A Nonverbal Communication Approach* . United Kingdom: University of Arizona Press.

[cit0035] Razzaghipour, A. , Ashrafi, M. , & Mohammadzadeh, A. (2024). A review of auditory attention: Neural mechanisms, theories. *Indian Journal of Otolaryngology and Head & Neck Surgery* , *76* , 2250–2256. 10.1007/s12070-023-04373-1 38883545 PMC11169100

[cit0036] Sathyanarayanan, H. , & Caldas, L. (2025). From challenges to innovations: Expert insights in pediatric healthcare design. *HERD: Health Environments Research & Design Journal* , *18* (4), 180–198. 10.1177/19375867251353733 40708309 PMC12460912

[cit0037] Sevón, E. , Mustola, M. , Siippainen, A. , & Vlasov, J. (2023). Participatory research methods with young children: A systematic literature review. *Educational Review* , *77* , 1–19. 10.1080/00131911.2023.2215465

[cit0038] Spencer, C. , & Blades, M. (2006). Children and their Environments: Learning, Using and Designing Spaces 10.1017/CBO9780511521232

[cit0039] Ulrich, R. S. (1991). Effects of interior design on wellness: Theory and recent scientific research. Journal of health care interior design. *Symposium on Health Care Interior Design* , 97–109. 10123973

[cit0040] Ulrich, R. S. (2001). Effects of healthcare environmental design on medical outcomes. In A. Dalani (Ed.), *Design and Health: The Therapeutic Benefits of Design* (pp. 49–59). Swedish Building Council.

[cit0041] Ulrich, R. S. , Zimring, C. , Zhu, X. , DuBose, J. , Seo, H. B. , Choi, Y. S. , Quan, X. , & Joseph, A. (2008). A review of the research literature on evidence-based healthcare design. *HERD* , *1* (3), 61–125. 10.1177/193758670800100306 21161908

[cit0042] Vygotsky, L. S. (2004). Imagination and creativity in childhood. *Journal of Russian & East European Psychology* , *42* (1), 7–97. 10.1080/10610405.2004.11059210

[cit0043] Watts, R. , & Wilson, S. (2009). Impact of the physical environment in paediatric hospitals on health outcomes: A systematic review. *JBI Library of Systematic Reviews* , *7* (20), 908–941. 10.11124/jbisrir-2009-201 27819847

[cit0044] Williams, T. , Ward, K. , Egli, V. , Mandic, S. , Pocock, T. , Clark, T. C. , & Smith, M. (2022). More-than-human: A cross-sectional study exploring children’s perceptions of health and health-promoting neighbourhoods in Aotearoa New Zealand. *International Journal of Environmental Research and Public Health* , *19* , 16968. 10.3390/ijerph192416968 36554849 PMC9779710

[cit0045] Yu, C. , Wong, E. , Gignac, J. , Walker, M. , & Ross, T. (2024). A scoping review of pediatric healthcare built environment experiences and preferences among children with disabilities and their families. *HERD: Health Environments Research & Design Journal* , *17* (2), 309–325. 10.1177/19375867231218035 38130020 PMC11080387

[cit0046] Zhang, M. , & Wyness, M. (2024). Children’s participation in decisions affecting their school life in China: Towards children’s subjective well-being in school. *Child Indicators Research* , *17* , 1941–1967. 10.1007/s12187-024-10151-4

