# Supplementary material for: Children in non-clinical healthcare environment: insights from a multi-method qualitative approach
Source: Int J Qual Stud Health Well-being. 2026 Aug 27;21(1):2721235. doi: 10.1080/17482631.2026.2721235 (PMC13523120; doi:10.1080/17482631.2026.2721235)
Supplement: Supplementary file 1 [file ZQHW_A_2721235_SM2990.docx]

**Supplementary Information-1(SI-1)**

**Overview of the physical settings**

**Title: Children in non-clinical healthcare environment: Insights from a multi-method qualitative study**

**Aim**:

To explore children’s behaviors and experiences within non-clinical healthcare settings, using a multi-method qualitative approach informed by Supportive Design Theory.

| Major category | | Enclosures | | | Fenestrations | | | Accessories | | |
| --- | --- | --- | --- | --- | --- | --- | --- | --- | --- | --- |
| Minor category | | Wall | Floor | Ceiling | Windows | Doors | Openings | Aesthetics | Furnishings | Fixtures |
| Characteristics/ attributes | | Height, finish, color, geometry, levels, pattern | | | Type, height, width, material, location | | | Artwork, installations, materials, texture, color, size, shape, pattern, location. | | |
| Setting 1 | Play area | - Wall - Solid wall- Plastered finish, height 300 cms, pale yellow in color; Partition wall – wooden, height 90 cms, pale yellow color, smooth surface, - Floor – vinyl flooring, non-geometrical pattern, vibrant red, blue and green colors, non-slippery surface, one level - Ceiling- Grid pattern Gypsum board with aluminum frames, AC outlet, light and fans. | | | - Windows – Sliding, Aluminum frame with glass, Sill level – 170 cms, placed at regular intervals – 3 in number - No doors and openings present. | | | - Play equipment: Slide- Made of plastic, 1 in number, height 90 cms, colors- Colors- orange, blue, yellow, smooth edges. - Swing: Made of steel, four poles with a swing in the middle, height of 210 cms, seats on two sides of the swing, metal plate as floor, swing hung from horizontal frame with chains, Vibrant red color. - No artwork and installations present. - No furniture was present. | | |
| Setting 2 | Waiting area | - Walls: Solid wall- Plastered finish, height 300 cms, Pale yellow paint color, Wall with posters and unit details, adjacent to play area. Wall lights- tube light and CFL bulbs; Notice board on wall: Information about department units & doctors and other hospital information. - Floor: Vitrified tiles, grid of 60 cms x 60 cms, white color, glossy finish, slippery, one level. - Ceiling: Grid pattern Gypsum board with aluminum frames, AC outlet, light and fans; Signages hung from ceiling | | | - Windows – Sliding, Aluminum frame with glass, Sill level – 170 cms, placed at regular intervals – 3 in number - Doors: Aluminum-glass door, aluminum frame 210 cms with glass shutter to 120 cms in height; Duct door: PVC door, wood finish, steel hinges and handles, 30 cms above FFL, 150 cms in height; door to Consultation room: 210 cms in height, wooden door, single shutter | | | - Aesthetic: Poster of fire-extinguisher, - Furniture: Reception counter- height 110 cms, laminated (Veneer) finish, brown + beige color, Geometric shape, sharp edges, immovable/fixed counter; Chairs- Waiting area: Metal chairs- glossy finish, Perforated, 60 cm wide, grey & black color, combination of 3 seats fixed in floor, Immovable in nature; Reception chairs: Wood & metal, cushioned fabric, brown color, movable. - Fire-extinguisher cylinder: Red color, hung on wall near duct door. - Suggestion box: Metal box 120 cms in height from FFL, white in color, glossy finish, hung on wall - Posters / information banners: On plastered walls, on Glass doors, on column, brown + beige + white color, white font color, COVID-19 poster etc. - Baby feeding cubicle: 250 cms in height, temporary movable structure, made of metal walls, semi-circular in form. | | |
| Setting 3 | Corridor | - Wall: Plastered pale yellow wall, glossy finish. Mural art of cartoon characters height 7’. - Floors: Vitrified tiles, 2’ x 2’ size, Uniform level - Ceiling: White ceiling, Gypsum board with aluminum frames Light fixtures on ceiling, Grid of 600mm x 600mm | | | - Door: Aluminum-glass door; Aluminum shutter till 4 ft. ht; Glass above 4’ till 7’. - Duct door: behind the corridor seating; brown color, metal door. - No windows were present | | | - Chairs: Metal chairs- glossy finish; Steel chairs – grey color; Immovable. - Guard rail: Spans on full length of mural wall, wooden, 1’6” ht. from FFL; smooth surface and edges | | |
| Setting 4 | Lobby | - Wall: Plastered pale yellow wall, glossy finish. Colorful mural art abstract design depicting a mother holding a child. Height- 7’ - Floors: Vitrified tiles, 2’ x 2’ size, Uniform level - Ceiling: White ceiling, Gypsum board with aluminum frames Light fixtures on ceiling, Grid of 600mm x 600mm | | | - Windows- Sliding Glass shutters, Aluminum panels. - Doors-Shuttered doors, Single shutter, Wood finish | | | - Reception counter: 4’6” high, Laminated (Veneer) finish, brown colour, Geometric shape, sharp edges - Chairs: Metal chairs- glossy finish Steel chairs – grey color, Immovable - Advertisements Standees- Digital Display standee 7’ tall, 2’6” wide, Blinks for Ads, Light always ON; Printed Standee 6’ tall, 1’ wide Cylindrical. | | |
