# Supplementary material for: Children in non-clinical healthcare environment: insights from a multi-method qualitative approach
Source: Int J Qual Stud Health Well-being. 2026 Aug 27;21(1):2721235. doi: 10.1080/17482631.2026.2721235 (PMC13523120; doi:10.1080/17482631.2026.2721235)
Supplement: Supplementary file 2 [file ZQHW_A_2721235_SM2991.docx]

**Supplementary Information-4 (SI-4)**

**Template of behavioral mapping of children's behaviors**

**Title: Children in non-clinical healthcare environment: Insights from a multi-method qualitative study**

**Aim**:

To explore children’s behaviors and experiences within non-clinical healthcare settings, using a multi-method qualitative approach informed by Supportive Design Theory.

| Behavioral mapping of children's behaviors (Frequency Count- FC) | | | |
| --- | --- | --- | --- |
| Date: __________ | Setting name: __________ | Start Time: __________  End Time: __________ | |
| Children's behaviors | **Tally Marks (FC)** | **Total count** |  |
| Playing (P)  Child engages with toys, play equipment, environmental features, or self-directed play activities. |  |  |  |
| Observing (O)  Child visually observes people, objects, displays, screens, environmental features, or surrounding activities without direct interaction. |  |  |  |
| Jumping (J)  Child performs repeated upward body movements involving both feet leaving the ground. |  |  |  |
| Touching (T)  Child intentionally touches walls, furniture, toys, displays, handrails, or other environmental elements. |  |  |  |
| Standing (St)  Child remains upright without substantial movement or engagement in another primary behavior. |  |  |  |
| Climbing (C)  Child climbs onto, over, or along furniture, play structures, seating elements, partitions, or other environmental features. |  |  |  |
| Sitting (Stt)  Child remains seated on chairs, benches, floors, or other surfaces. |  |  |  |
