# Supplementary material for: Children in non-clinical healthcare environment: insights from a multi-method qualitative approach
Source: Int J Qual Stud Health Well-being. 2026 Aug 27;21(1):2721235. doi: 10.1080/17482631.2026.2721235 (PMC13523120; doi:10.1080/17482631.2026.2721235)
Supplement: Supplementary file 3 [file ZQHW_A_2721235_SM2992.docx]

**Supplementary Information-2 (SI-2)**

**Sample of an observation map**

**Title: Children in non-clinical healthcare environment: Insights from a multi-method qualitative study**

**Aim**:

To explore children’s behaviors and experiences within non-clinical healthcare settings, using a multi-method qualitative approach informed by Supportive Design Theory.

Sample of an observation map of setting- play area
