# Supplementary material for: Children in non-clinical healthcare environment: insights from a multi-method qualitative approach
Source: Int J Qual Stud Health Well-being. 2026 Aug 27;21(1):2721235. doi: 10.1080/17482631.2026.2721235 (PMC13523120; doi:10.1080/17482631.2026.2721235)
Supplement: Supplementary file 4 [file ZQHW_A_2721235_SM2993.docx]

**Supplementary Information-3 (SI-3)**

**Verbal Elicitation guide**

**Title: Children in non-clinical healthcare environment: Insights from a multi-method qualitative study**

**Aim**:

To explore children’s behaviors and experiences within non-clinical healthcare settings, using a multi-method qualitative approach informed by Supportive Design Theory.

M / F:

Age:

Setting name:

**Verbal consent:**

Would you like to participate in this interview?

- Verbal Consent was obtained from the study participant ☐
- Verbal Consent was NOT obtained from the study participant ☐

**Probe questions:** (Based on the context & physical elements present in the setting)

The prompt questions:

• Would you like to draw me a picture?

• What do you like in this place?

• Would you like to draw something that you like in this place?

• Can you tell me about your experiences at the hospital.

• Would you like to draw something that you don't like in this place?

• What is important for you when you are at the hospital? What do you like here?

• What is that object/corner in this place that you don't want to see on your next time, if any?
